# Loss of CASQ2 promotes vascular smooth muscle cell phenotypic switching in aortic dissection uncovered by integrated single-cell transcriptomics

**DOI:** 10.1186/s12920-026-02410-w

**Published:** 2026-06-13

**Authors:** Kaiyan Chen, Meixiang Wang, Zhiyuan Zhang, Yingquan Chen, Hui Zhu, Ting Yang, Yuan Xia, Maomao Guo, Junchao Li

**Affiliations:** 1 Department of Cardiology, The Affiliated Taizhou People’s Hospital of Nanjing Medical University, Taizhou School of Clinical Medicine, Nanjing Medical University, Taizhou, 225300 China; 2 Department of Hematology, The Affiliated Taizhou People’s Hospital of Nanjing Medical University, Taizhou School of Clinical Medicine, Nanjing Medical University, Taizhou, 225300 China; 3 Department of Urology, The Affiliated Taizhou People’s Hospital of Nanjing Medical University, Taizhou School of Clinical Medicine, Nanjing Medical University, Taizhou, 225300 China; 4 Graduate School, Nanjing Medical University, Nanjing, 210029 China

**Keywords:** Aortic dissection, CASQ2, Vascular smooth muscle cells, Phenotypic switching, Single-cell RNA sequencing

## Abstract

**Background:**

Aortic dissection (AD) is a life-threatening cardiovascular disease with limited effective therapeutic options. Phenotypic switching of aortic vascular smooth muscle cells (VSMCs) from a contractile to a pathological state is a critical driver of AD progression. However, the molecular regulators governing this transition remain incompletely understood. This study aimed to identify a critical regulator of VSMC phenotypic switching in AD and to investigate its underlying mechanisms and translational relevance.

**Methods:**

Single-cell RNA sequencing (scRNA-seq) and bulk RNA sequencing datasets obtained from the Gene Expression Omnibus (GEO) database were integrated for analysis. To enhance robustness and reduce dataset-specific bias, weighted gene co-expression network analysis (WGCNA) was performed in both aortic aneurysm (AA) and AD cohorts to identify gene modules associated with VSMC function. Overlapping VSMC-related modules were intersected to define candidate genes. Key regulators were further prioritized in AD datasets using LASSO regression and validated by single-cell RNA sequencing (scRNA-seq) pseudotime trajectory analysis to delineate dynamic gene expression during VSMC phenotypic switching. Functional validation was conducted in an angiotensin II (Ang II)-induced experimental AD mouse model with AAV9-mediated CASQ2 overexpression, as well as in primary VSMCs isolated from the abdominal aorta of mice and treated with Ang II in vitro. Aortic morphology, medial calcification, VSMC phenotypic markers, intracellular Ca^2+^ homeostasis, and endoplasmic reticulum (ER) stress signaling were systematically evaluated.

**Results:**

WGCNA identified a conserved gene module comprising 64 genes associated with VSMC phenotypic transition. Integrative LASSO and scRNA-seq analyses prioritized CASQ2 as a key gene with reduced expression in AD VSMCs. Pseudotime analysis revealed progressive downregulation of CASQ2 during osteochondrogenic phenotypic switching. In AngII-induced experimental AD mice, CASQ2 expression was decreased in the aortic media and accompanied by enhanced medial calcification. In vivo, Ang II infusion markedly reduced aortic CASQ2 expression and induced medial dilation and calcification, accompanied by increased mortality. AAV9-mediated CASQ2 overexpression significantly improved survival, attenuated aneurysmal enlargement, reduced vascular calcification and partially restored contractile marker expression. In vitro, Ang II stimulation decreased CASQ2 levels in primary VSMCs, promoted osteochondrogenic marker expression, and induced intracellular Ca²⁺ overload. CASQ2 overexpression restored calcium-release complex components, suppressed cytosolic Ca²⁺ elevation, and markedly reduced ER stress activation.

**Conclusions:**

CASQ2 may represent a VSMC-enriched functional regulator involved in AD-associated phenotypic switching through calcium homeostasis and ER stress. These findings suggest that CASQ2 represents a potential therapeutic target in AD. Further studies are needed to determine its disease specificity and translational relevance.

**Supplementary Information:**

The online version contains supplementary material available at 10.1186/s12920-026-02410-w.

## Introduction

Acute Stanford type A aortic dissection (ATAAD), involving the ascending aorta, is a catastrophic cardiovascular emergency characterized by abrupt intimal disruption and separation of the aortic wall layers, leading to false lumen formation and rapid medial destabilization [1]. As the most lethal form of thoracic aortic dissection, ATAAD requires urgent surgical intervention and remains associated with substantial early mortality. Despite advances in surgical techniques and perioperative care, aortic dissection (AD) continues to pose significant clinical challenges. Current medical management of AD is largely limited to heart rate and blood pressure control and does not directly target the molecular mechanisms driving medial degeneration and structural failure [2]. This therapeutic limitation reflects an incomplete understanding of the pathobiology underlying ATAAD.

The fundamental pathological hallmark of AD is structural failure of the aortic media, characterized by medial degeneration and compromised extracellular matrix integrity, processes that are closely linked to maladaptive phenotypic switching and dysfunction of vascular smooth muscle cells (VSMCs) within the medial layer [3, 4]. VSMCs are the principal constituents of the aortic media, where they maintain vascular tone and structural integrity through their contractile properties. Under physiological conditions, VSMCs exist in a quiescent, highly differentiated contractile phenotype. However, in response to injury or hemodynamic stress, these cells exhibit remarkable plasticity, undergoing a process termed phenotypic switching. During this transition, VSMCs shift from a contractile phenotype into diverse pathological phenotypes, including synthetic, extracellular matrix (ECM) remodeling and osteochondrogenic phenotype [5, 6]. While this phenotypic switching is known to facilitate ECM remodeling and medial thinning observed in AD, the specific molecular triggers that initiate and govern this transition remain incompletely defined [7].

Identifying the key drivers of VSMC phenotypic switching remains a central challenge in AD research. The acute and catastrophic nature of AD often obscures early regulatory events, as transcriptomic profiles are dominated by secondary inflammatory and stress responses [6, 8, 9]. To address this limitation, we incorporated aortic aneurysm (AA) datasets into our initial screening framework. Although AA and AD differ clinically, both share a common pathological substrate of medial degeneration characterized by loss of the VSMC contractile phenotype and progressive structural destabilization of the aortic wall [3, 4]. Under physiological conditions, VSMCs maintain vascular integrity through a differentiated contractile state. However, pathological stress induces phenotypic switching toward synthetic and other maladaptive states, contributing to medial degeneration in both AA and AD [10–12].

Based on this shared biological foundation, we applied weighted gene co-expression network analysis (WGCNA) to integrated AA and AD transcriptomic datasets to identify conserved regulatory modules associated with VSMC phenotypic modulation. Although AA and AD are clinically distinct entities, both share pathological features of aortic medial degeneration, extracellular matrix remodeling, and VSMC phenotypic modulation. Therefore, AA datasets were incorporated not to define AD-specific biomarkers, but to identify conserved aortic disease-associated VSMC transition-related modules. Candidate genes from overlapping AA and AD modules were subsequently evaluated in AD datasets and experimental models to investigate their functional relevance to AD-associated VSMC remodeling. By combining co-expression analysis with single-cell trajectory reconstruction, we delineated candidate regulators that govern the transition of VSMCs from a contractile phenotype toward pathological states in AD.

## Materials and methods

### Datasets and preprocessing

Transcriptomic datasets for AD and AA were retrieved from the NCBI Gene Expression Omnibus (GEO) database (https://www.ncbi.nlm.nih.gov/geo/). For the AD analysis, three bulk RNA-sequencing datasets—GSE190635, GSE52093, and GSE153434—were utilized, comprising a total of 40 samples (21 AD patients and 19 healthy controls). In addition, a single-cell RNA-sequencing (scRNA-seq) dataset, GSE213740, was incorporated to characterize the cellular landscape of AD, which included 3 healthy control samples and 6 AD samples. For the AA analysis, to enhance statistical power and ensure the reliability of identified gene modules, three independent bulk transcriptomic datasets, including GSE57691, GSE7084, and GSE47472, were integrated, resulting in a total of 85 samples after rigorous quality control, including 65 AA patients and 20 healthy controls. Furthermore, patients with tricuspid aortic valve-associated aortopathy (TAV) were systematically excluded from all datasets prior to downstream analyses. Detailed information regarding the samples and datasets for both diseases is provided in Table 1.

**Table 1:** Metadata of the GEO datasets for AA and AD cohorts

GEO series	Research Focus	Data Type	Control (*n*)	Disease (*n*)	Total (*n*)
GSE190635	Acute Stanford Type A Aortic Dissection (ascending aorta)	Bulk mRNA sequencing	4	4	8
GSE52093	Acute Stanford Type A Aortic Dissection (ascending aorta)	Bulk mRNA sequencing	5	7	12
GSE153434	Acute Stanford Type A Aortic Dissection (ascending aorta)	Bulk mRNA sequencing	10	10	20
GSE57691	Abdominal Aortic Aneurysm	Bulk mRNA sequencing	10	49	59
GSE7084	Abdominal Aortic Aneurysm	Bulk mRNA sequencing	2	2	4
GSE47472	Abdominal Aortic Aneurysm	Bulk mRNA sequencing	8	14	22
GSE213740	Acute Stanford Type A Aortic Dissection (ascending aorta)	Single-cell RNA-seq	3	6	9

### Construction of a co-expression network and identification of modules

A systematic biological approach was used with WGCNA in order to identify co-expression modules associated with disease status and VSMC phenotypic transition.

### Functional enrichment analysis

Gene Ontology (GO) and Kyoto Encyclopedia of Genes and Genomes (KEGG) pathway enrichment analyses were carried out to characterize the biological function and mechanism of the related pathogenic genes. A threshold of *P* < 0.05 was considered to indicate significant enrichment. Moreover, bubble diagrams and chord diagrams were used to visualize the results of functional enrichment analysis.

### Machine learning

The least absolute shrinkage and selection operator (LASSO) algorithm, which uses logistic regression to filter variables to enhance predictive performance, was applied to screen for candidate biomarkers via the “glmnet” package. LASSO regression was applied to prioritize candidate genes from the overlapping module genes for subsequent AD-focused single-cell and experimental validation.

### Single-cell sequencing analysis

The scRNA-seq data analysis was conducted using Seurat (v. 4.3.0). Raw scRNA-seq count matrices were processed using standard preprocessing workflows. Cells were subjected to stringent quality control filtering to ensure high-confidence single-cell profiles. Cells with mitochondrial gene expression exceeding 15% of total transcripts were removed to exclude stressed or low-quality cells [13]. To control for transcript complexity and potential multiplet contamination, only cells expressing between 300 and 5,000 genes were retained for downstream analyses, thereby removing low-coverage droplets and extreme outliers [14].To further reduce technical noise arising from barcode collisions, putative doublets were computationally identified using DoubletFinder, which generates artificial doublets and distinguishes them from singlets based on neighborhood relationships in principal component space.

We then analyzed the remaining cells downstream, performed normalization, selected the highly variable genes, completed scaling, and conducted principal component analysis (PCA). The “harmony” package was used to eliminate the batch effect of our combined scRNA-seq data. As a following step to harmonic integration, we performed uniform manifold approximation and projection (UMAP) using RunUMAP. Clusters of cells were identified using FindNeighbors and FindClusters (resolution = 0.2). Default parameters were used for all standard analysis procedures.

By identifying the DEGs in each cluster, we ensured the accuracy of cell annotation. Based on the published literature, we made a comprehensive judgment regarding which cell type to use. Cell types were annotated according to the expression of established canonical marker genes, including epithelial cells (EPCAM, KRT8, KRT18), T cells (CD3D, CD2, CD3E), B cells (CD19, CD79A, MS4A1 [CD20]), plasmablasts (JCHAIN, IGHG4, MZB1), myeloid cells (CD68, LYZ, S100A9), endothelial cells (PECAM1, VWF, ADGRL4), and fibroblasts (PDGFRA, DCN, COL1A1). Differentially expressed genes (DEGs) were identified using the Wilcoxon rank-sum test with a Bonferroni-corrected P-value < 0.05.

### Pseudotime trajectory analysis of potential VSMC biomarkers for AD progression

The R package “monocle” version 2.14.0 under default settings was used to conduct pseudotime trajectory analysis. As part of the pseudotime trajectory, dimension reduction and cell ordering were completed via the dynamic dimension reduction tree method. Branch expression analysis modeling was used for branch analysis. As a result of the distinct patterns of gene expression changes leading to the different cell states, 4 gene blocks were selected to present the genes that exhibited significant changes (acquired from the first 100 genes demonstrating significant changes based on the P value) at a branch point.

### Western blotting

Immunoblotting protein was extracted with radioimmunoprecipitation assay (RIPA) buffer. In this experiment, the protein was separated by sodium dodecyl sulfate-polyacrylamide gel electrophoresis (SDS-PAGE) and then transferred onto a polyvinylidene difluoride (PVDF) membrane. As a blocking step, the membranes were incubated with 5% nonfat milk for 5 min before being incubated with the primary antibodies and secondary antibodies. The antibodies used were anti-CASQ2 (1:1,000; cat. no. 18422-1-AP; Proteintech), anti-RUNX2 (1:1,000; cat. no. A11753; Abclonal), anti-SMA (1:1,000; cat. no. 14395-1-AP; Proteintech), anti-BMP-2 (1:1,000; cat. no. 66383-1-Ig; Proteintech), anti-SERCA2 (1:1,000; cat. no. 27311-1-AP; Proteintech), anti-Triadin (1:1,000; cat. no. MA3-927; Invitrogen, Thermo Fisher Scientific, Waltham, MA, USA), anti-Junctin/ASPH (1:1,000; cat. no. 14116-1-AP; Proteintech), and anti-GAPDH (1:2,000; cat. no. AF7021l; BioReagents, Thermo Fisher Scientific).

### Immunohistochemical staining

In this study, serial sections underwent immunohistochemical staining. Tissue sections were stained with the EnVision+ system (Agilent, Santa Clara, CA, USA) as per the manufacturer’s instruction. The tissue was sectioned using formalin-fixed, paraffin-embedded slides, and the paraffin was removed using xylene (Thermo Fisher Scientific). As a means to unmask the antigens, citrate buffer (Thermo Fisher Scientific) was applied. A 3%-hydrogen peroxide solution in methanol was used to quench endogenous peroxidases. An antibody against anti-CASQ2 (1:100; cat. no. 18422-1-AP; Proteintech) was used to stain the samples. Mayer’s hematoxylin (Agilent) was used for counterstaining. Immunohistochemical staining was used for representative visualization of CASQ2 expression in aortic tissues and was not included in quantitative statistical analysis.

### Animal study

Animal experiments were conducted in accordance with the ARRIVE guidelines (Animal Research: Reporting of In Vivo Experiments) for the care and use of laboratory animals. The protocols involving animals were approved by the Animal Care and Use Committee of Nanjing Medical University (No. IACUC-2501025). C57BL/6J male mice were obtained from and kept at the Animal Core Facility of Nanjing Medical University (Nanjing, China) at a constant humidity and temperature. Water and food were provided to all mice without restriction. During all experimentation, mice were kept under controlled conditions with a strict 12-hour cycle of light and dark. The mice were anesthetized with 1–2% isoflurane and sacrificed via cervical dislocation at the end of the study. Male C57BL/6J mice aged 3 weeks were used to establish an Ang II-induced experimental AD model as previously described. The use of young mice was based on prior pharmacologically induced aortic aneurysm/dissection models, in which juvenile mice exhibit increased susceptibility to aortic wall destabilization and develop reproducible aortic lesions [15, 16]. Ang II was delivered at a rate of 1,000 ng/kg/min for 4 weeks using osmotic minipumps. Although this model does not fully reproduce the complexity of human AD, it recapitulates several relevant pathological features, including aortic dilation, medial degeneration, VSMC phenotypic switching, and vascular calcification. Mice were randomly assigned to the following groups: AAV9-Control + saline group (*n* = 6), AAV9-CASQ2 + saline group (*n* = 6), AAV9-Control + Ang II group (*n* = 6), and AAV9-CASQ2 + Ang II group (*n* = 6). Mice received adeno-associated vectors serotype 9 (AAV9) expressing Casq2 (AAV9-Casq2) or an empty polylinker (AAV9-Control) via tail vein injection, with each mouse receiving 100 µl of phosphate-buffered saline (PBS) containing 2 × 10^11^ vector genomes (vg).

### Alizarin red staining

Paraffin sections were baked at 60–65 °C for 1–2 h to ensure firm adhesion of the tissue sections to the glass slides. The sections were then immersed successively into xylene I and xylene II for 10 min each in order to completely remove the paraffin and expose the tissues. Following this, the dewaxed sections were transferred to absolute ethanol, 95% ethanol, 80% ethanol, and 70% ethanol in sequence, with immersion in each being conducted for 3 to 5 min at a time. After a three-time thorough rinsing of the sections with distilled water for 1 min each time, the tissues were hydrated. The hydrated sections were placed in alizarin red staining solution and stained for 15–30 min at room temperature. The staining progress was observed under microscopy. The areas rich in calcium salts gradually turned red. After staining was completed, the sections were dipped in distilled water three to five times for 1 min each time to differentiate and wash away excess staining solution. This was done to make the background clear until the background color of the sections became lighter and the red color of the calcium salt-positive regions became visible.

### Immunofluorescence staining

Paraffin sections were baked at 60–65 °C for 1–2 h to ensure firm adhesion of the tissue sections to the glass slides. The sections were deparaffinized in xylene I and xylene II for 10 min each and rehydrated through a graded ethanol series of absolute ethanol, 95% ethanol, 80% ethanol, and 70% ethanol for 3–5 min each. After rinsing with PBS three times for 5 min each, antigen retrieval was performed using citrate antigen retrieval buffer. The sections were then allowed to cool naturally to room temperature and washed with PBS three times. After blocking with blocking solution at 37 °C for 30–60 min, the sections were incubated with primary antibodies against α-SMA (1:100; cat. no. 67735-1-Ig; Proteintech), RUNX2 (1:100; cat. no. A11753; Abclonal), and CASQ2 (1:100; cat. no. 18422-1-AP; Proteintech). After washing, the sections were incubated with Alexa Fluor 555 goat anti-mouse IgG (H + L) and Alexa Fluor 488 goat anti-rabbit IgG (H + L) secondary antibodies. Nuclei were counterstained with DAPI.

### Isolation, primary culture, and treatment of mouse abdominal aortic VSMCs

Mouse abdominal aortas were dissected and cultured in cold Dulbecco’s Modified Eagle Medium (DMEM) with antibiotics (100 U/mL penicillin and 100 mg/mL streptomycin) in sterile dishes. After the connective tissue was removed, the aorta was pinned on a silicone-covered preparation dish. The endothelium was gently removed with curved forceps after the vessel was cut longitudinally. After stripping off the medial layer in small pieces, a T25 culture flask was placed upright in a tissue incubator, and the specimens were incubated. Every 48 h, the flask was transferred horizontally, and the medium was changed every 48 h. On days 7 and 10, media explants were removed with single cells plated into 35-mm Petri dishes at low densities. The abdominal aortic VSMCs were plated in 6-well plates or 24-well plates and cultured in a 5% CO2 incubator at 37 ℃ for subsequent studies. VSMCs were treated with 100 nM Ang II for 24 h to induce phenotypic switching and cellular stress. VSMCs were transduced for 48 h with recombinant adenovirus encoding CASQ2 (Ad-CASQ2) to overexpress CASQ2 or with adenovirus encoding GFP (Ad-GFP).

### Ca^2+^ imaging in VSMCs

Rhod-2/AM Ca^2+^ indicators were used to monitor cytosolic Ca^2+^. The cultured cells were washed 3 times with buffer. Rhod-2/AM working solution was applied to cover the cells. The cells were incubated for 15–60 min at 37 ℃ after which the Rhod-2/AM working solution was removed. Probe-free buffer was applied at least three times to remove all the remaining Rhod-2/AM working solution. Once the cells were covered with buffer, they were incubated at 37 ℃ for about 20–30 min. Ca^2+^ fluorescence was visualized with a fluorescence microscope (Carl Zeiss, Oberkochen, Germany).

### Quantification of experimental data

Quantification of Western blotting, immunofluorescence staining, Alizarin Red staining, and calcium imaging was performed using ImageJ software. Immunohistochemical staining was used for representative visualization of CASQ2 expression in aortic tissues and was not included in quantitative statistical analysis. For Western blotting, band intensities were measured after background subtraction. The intensity of each target protein band was normalized to GAPDH, and the normalized values were expressed relative to the corresponding control group. For immunofluorescence staining, images were acquired from comparable regions of the aortic media or cultured VSMCs under identical acquisition settings within each staining batch, including the same microscope, objective magnification, exposure time, illumination intensity, camera gain, and image resolution. Mean fluorescence intensity was measured in selected regions of interest after background subtraction.

For Alizarin Red staining, calcification was quantified using threshold-based image analysis. The Alizarin Red-positive area was measured and expressed as the percentage of positively stained area relative to the total medial area in the same section. For calcium imaging, Rhod-2 fluorescence intensity was measured in VSMCs after background subtraction. The fluorescence intensity was normalized to the control group and used to reflect relative intracellular Ca²⁺ levels.

### Statistical analysis

Statistical analyses were performed using R software, SPSS 23.0, and GraphPad Prism 8.0, as appropriate. Quantitative experimental data are presented as mean ± SEM. Data distribution was assessed using the Shapiro–Wilk test before statistical comparison. For comparisons between two groups, an unpaired two-tailed Student’s t-test was used for normally distributed data, whereas the Mann–Whitney U test was used for non-normally distributed data. For comparisons among more than two groups, one-way analysis of variance (ANOVA) followed by Tukey’s post hoc test was used for normally distributed data, whereas the Kruskal–Wallis test followed by Dunn’s multiple-comparison test was used for non-normally distributed data. Kaplan–Meier survival curves were compared using the log-rank test. For transcriptomic analyses, differential expression analysis and enrichment analyses were performed using the statistical methods implemented in the corresponding R packages. Multiple-testing correction was applied where appropriate, and adjusted P values or false discovery rate values were used to determine statistical significance. For scRNA-seq differential expression analysis, the Wilcoxon rank-sum test with Bonferroni correction was used. For GO and KEGG enrichment analyses, terms with P values or adjusted P values below the specified threshold were considered significant, as indicated in the corresponding analyses. The number of animals, independent biological replicates, independent cell experiments, and analyzed microscopic fields are indicated in the corresponding figure legends. A two-sided P value < 0.05 was considered statistically significant.

## Results

### Identification of conserved VSMC transition-related candidate genes in aortic disease

**Fig. 1:**
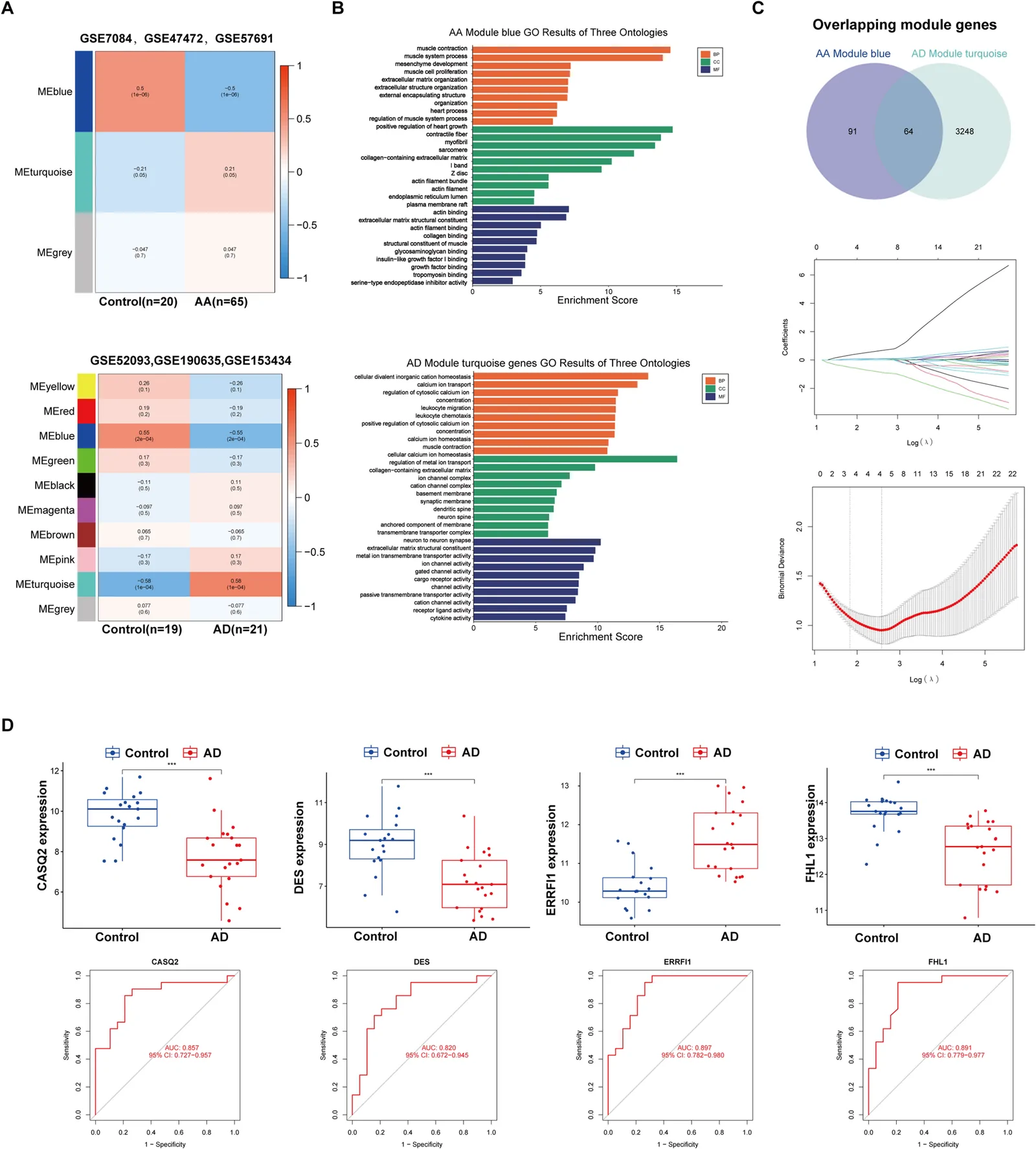
Identification of conserved VSMC-associated modules and candidate biomarkers in AD. **A** Module-trait relationship heatmaps of AA (GSE7084, GSE47472, GSE57691) and AD (GSE52093, GSE190635, GSE153434) datasets. Each cell displays the correlation coefficient and corresponding P value between module eigengenes and disease status. **B** GO enrichment analysis of genes within the AA blue module and AD turquoise module. Bar plots show significantly enriched biological processes (BP), cellular components (CC), and molecular functions (MF). **C** Venn diagram showing overlapping genes between the AA blue module and AD turquoise module. LASSO regression identified four candidate genes: CASQ2 (calsequestrin 2), DES (desmin), ERRFI1 (ERBB receptor feedback inhibitor 1), and FHL1 (four and a half LIM domains 1). **D** Differential expression analysis and diagnostic performance of CASQ2, DES, ERRFI1, and FHL1 in AD versus control samples. Upper panels: boxplots of gene expression. Lower panels: ROC curves with AUC values.Statistical significance for gene expression comparisons was determined using Student’s t-test or the Mann–Whitney U test, as appropriate. ROC curves were used to evaluate the discriminatory performance of candidate genes. GO and KEGG enrichment analyses were performed using the corresponding R packages, and terms with *P* < 0.05 were considered significant unless otherwise specified. (Data are presented as the mean ± SEM. *, *P* < 0.05; **, *P* < 0.01; ***, *P* < 0.001; ns, no significant difference.)

To identify key regulators of VSMC phenotypic transition while reducing dataset-specific bias, WGCNA was performed independently in both AA and AD transcriptomic datasets. This cross-pathology approach improved the robustness of module identification by capturing gene networks commonly associated with VSMC phenotypic transition across distinct aortic disease contexts. In the AD cohort, ten gene modules were identified using a soft-thresholding power of 4. Correlation analysis (Fig. 1A) showed that the turquoise module exhibited the strongest positive correlation with AD (3,312 genes; *r* = 0.58; *P* = 1 × 10^− 4^), whereas the blue module showed the strongest negative correlation (1,638 genes; *r* = − 0.55; *P* = 2 × 10^− 4^). In the AA cohort, the blue module displayed a significant negative correlation (155 genes; *r* = 0.5; *P* = 1 × 10^− 6^). GO enrichment (Fig. 1B) demonstrated that disease-associated modules identified in both AA and AD were enriched for biological processes related to VSMC phenotypic transition. In AA, the blue module was significantly enriched in GO terms related to the contractile function, including “muscle contraction”, “muscle system process”, “contractile fiber”, “myofibril”, and “sarcomere”, representing biological processes associated with VSMC contractile state. In AD, the turquoise module was enriched for GO categories such as “muscle contraction” and “collagen-containing extracellular matrix organization”, together with calcium-regulatory processes including “cellular calcium ion homeostasis”, “regulation of cytosolic calcium ion concentration”, and “calcium ion transport”. These enriched biological processes are consistent with previously reported roles of calcium regulation in VSMC phenotypic transition [17].

KEGG enrichment revealed that the AA blue module was primarily associated with “vascular smooth muscle contraction”, “actin cytoskeleton regulation”, and “focal adhesion”, highlighting pathways governing VSMC contractile function. In contrast, the AD turquoise module was enriched in “cytokine-cytokine receptor interaction”, “IL-17 signaling”, and “calcium signaling pathways”. Notably, both modules converged on cytoskeletal and calcium-related processes, supporting a shared molecular framework underlying VSMC phenotypic modulation in aortic pathology (Supplementary Fig. 1A). GO and KEGG enrichment of other module gene sets in both AA and AD showed limited enrichment in VSMC-related pathways (Supplementary Fig. 1B–C). Collectively, these findings indicate that independently derived AA and AD modules converge on biological processes involving VSMC functional remodeling and calcium-associated pathways.

Accordingly, intersecting the AA blue module and AD turquoise module enabled the identification of a shared gene set likely representing conserved molecular programs underlying VSMC phenotypic transition. A total of 64 overlapping genes were obtained (Fig. 1C). Candidate genes identified by LASSO regression included CASQ2 (*calsequestrin 2*), DES (*desmin*), ERRFI1 (*ERBB receptor feedback inhibitor 1*), and FHL1 (*four and a half LIM domains 1*).(Fig. 1C). As shown in Fig. 1D, the expression levels of *CASQ2*,* FHL1*, and *DES* were significantly lower in the AD group compared with controls, whereas *ERRFI1* expression was significantly higher in AD samples (Fig. 1D). Receiver operating characteristic (ROC) curve analysis demonstrated favorable diagnostic performance for all four genes, with area under the curve (AUC) values of 0.857 for *CASQ2*, 0.820 for *DES*, 0.897 for *ERRFI1*, and 0.891 for *FHL1* (Fig. 1D). These results suggest that the identified genes may have potential value as VSMC-enriched functional candidate genes for AD. We next examined whether the candidate genes were altered in AA, thereby assessing whether they reflected AD-specific changes or more general aortic disease-related remodeling. CASQ2 was significantly downregulated in AA compared with controls, while ERRFI1 and FHL1 were also reduced and DES showed a nonsignificant decreasing trend (Supplementary Fig. 3A). ROC analysis showed modest discriminatory performance in AA, with AUC values of 0.652 for CASQ2, 0.620 for DES, 0.683 for ERRFI1, and 0.713 for FHL1 (Supplementary Fig. 3B). These findings suggest that CASQ2 is involved in broader aortic disease-related VSMC remodeling and should be regarded as a VSMC transition-related candidate gene rather than an AD-specific diagnostic biomarker.

### ScRNA-seq profiling identifies CASQ2 as a key differentially expressed gene in AD-derived VSMCs

**Fig. 2:**
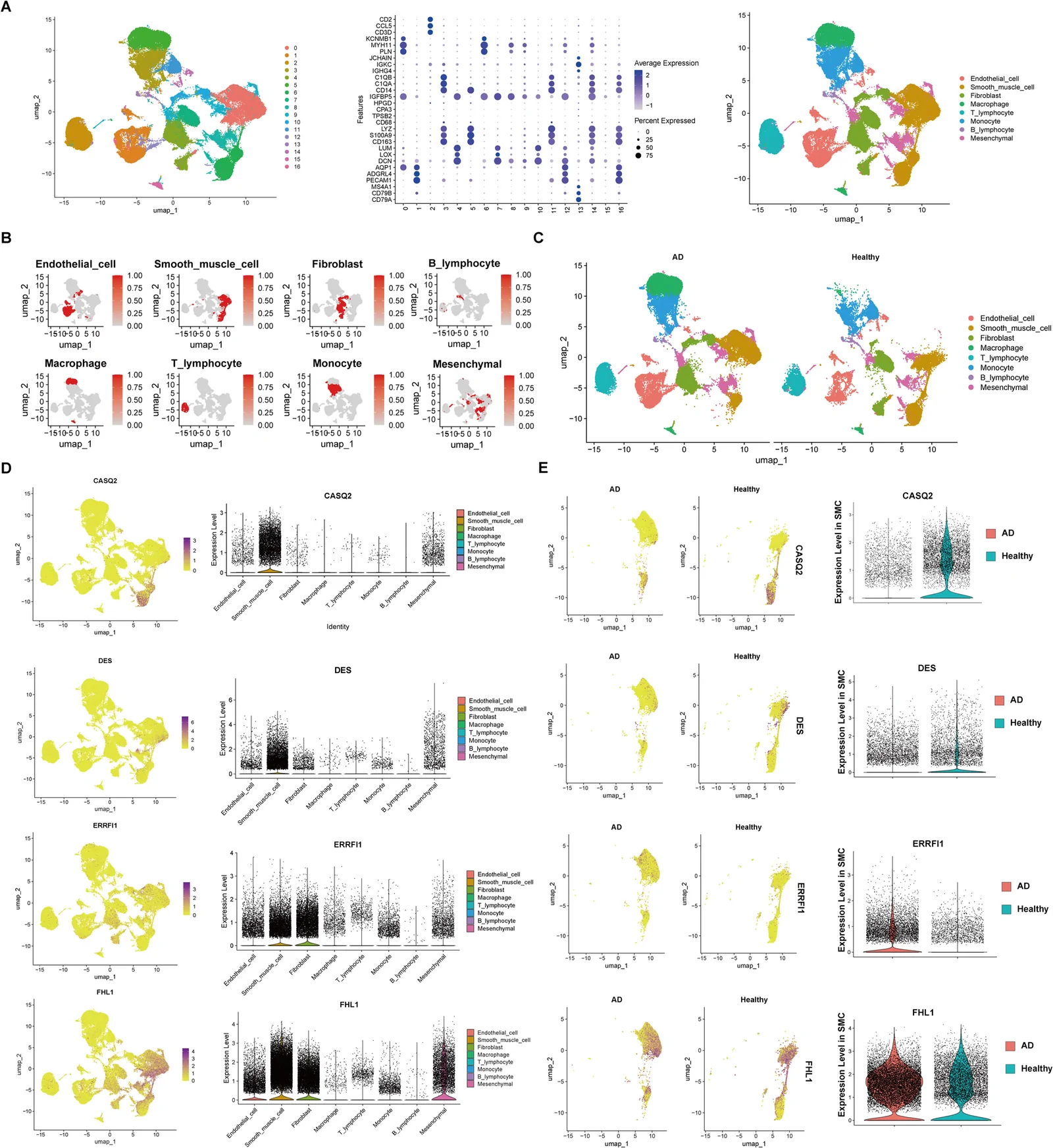
scRNA-seq characterization of normal aortic vascular tissue and aortic dissection vascular tissue. **A** UMAP visualization of integrated scRNA-seq data showing unsupervised clustering of cells from AD and healthy aortic tissues (left). Dot plot displaying canonical marker genes used for cell-type annotation across clusters (middle). UMAP colored by annotated cell types (right), including endothelial cells, smooth muscle cells (SMCs), fibroblasts, macrophages, T lymphocytes, monocytes, B lymphocytes, and mesenchymal cells. **B** Feature plots illustrating the spatial distribution of each annotated cell type on the UMAP embedding. **C** UMAP plots showing the distribution of major cell populations in AD and healthy samples separately, highlighting differences in cellular composition between groups. **D** Expression patterns of four key genes (CASQ2, DES, ERRFI1, and FHL1). Left: Feature plots showing gene expression across all cell populations. Right: Strip plots summarizing gene expression levels in each annotated cell type. **E** Expression of CASQ2, DES, ERRFI1, and FHL1 in SMCs from AD and healthy samples. Left: Feature plots displaying gene expression in SMCs for AD and healthy groups. Right: Violin plots comparing gene expression levels between AD and healthy SMCs. Differential expression analysis in scRNA-seq data was performed using the Wilcoxon rank-sum test with Bonferroni correction. (Data are presented as the mean ± SEM. *, *P* < 0.05; **, *P* < 0.01; ***, *P* < 0.001; ns, no significant difference.)

To elucidate the cell-specific molecular changes and identify key drivers of VSMC phenotypic switching in AD, we performed scRNA-seq and pseudotime analysis on human aortic tissues. We first processed and analyzed scRNA-seq data obtained from ascending aortic wall tissues of six patients with AD and three heart transplant donors. Quality control resulted in the selection of 17 principal components for further analysis via PCA. The UMAP dimensionality reduction method enabled us to identify nine distinct subsets of cells (Fig. 2A and B). Based on canonical marker genes, eight major cell types were annotated, including endothelial cells, smooth muscle cells (SMCs), fibroblasts, macrophages, T lymphocytes, monocytes, B lymphocytes, and mesenchymal cells (Fig. 2A). The dot plot of representative marker genes confirmed the reliability of cell-type annotation. Feature plots further demonstrated the specific spatial distribution of each cell population within the UMAP embedding (Fig. 2B). Comparative analysis between AD and healthy samples revealed alterations in cellular composition. Notably, immune-related cell populations, including macrophages and T lymphocytes, were relatively enriched in AD tissues, whereas the distribution pattern of SMCs exhibited marked changes (Fig. 2C), suggesting an inflammatory microenvironment and structural remodeling in AD.

The expression levels of four genes across different cell types are presented in Fig. 2D. The results showed that both *CASQ2* had higher expression levels in VSMCs than in other cell types, indicating that *CASQ2* was VSMC-specific high-expression genes. We obtained a Seurat matrix comprising 21,292 smooth muscle cells, including 8,623 normal VSMCs and 12,669 VSMCs from AD. The expression patterns of *CASQ2*,* DES*,* ERRFI1* and *FHL1* were analyzed and compared between AD and healthy samples (Fig. 2E). The findings suggested that *CASQ2* and *DES* had a lower expression in AD samples than in healthy samples. Meanwhile, the expression of *ERRFI1* was higher in AD samples whereas there was no significant difference of *FHL1* expression between the healthy and the AD samples.

### Pseudotime trajectory analysis links CASQ2 reduction to VSMC phenotypic switching

**Fig. 3:**
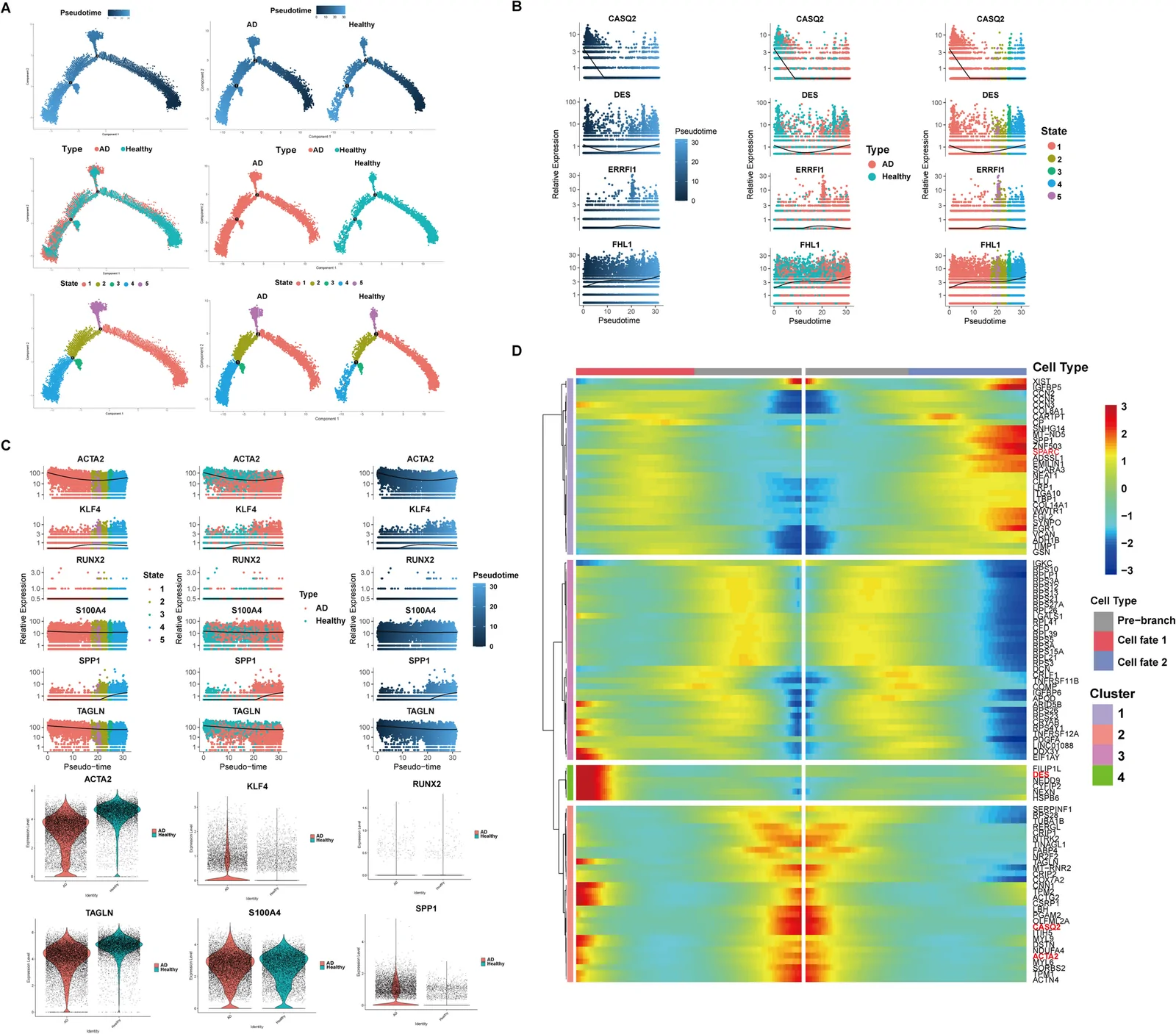
Pseudotime analysis of smooth muscle cells in samples from patients with AD and healthy controls. **A** Pseudotime trajectory plot, Type distribution trajectory plot and State distribution trajectory plot of healthy and AD samples. The color gradient from light blue to dark blue indicates pseudotime progression (from early to late), illustrating the distribution of samples/cells along the pseudotime trajectory. Red denotes the AD group, and turquoise denotes the Healthy group, showing the spatial distribution of the two groups along the trajectory. Colors represent State 1 to 5, illustrating the distribution of different states along the trajectory. **B** The expression levels of CASQ2, DES, ERRFI1 and FHL1 in the pseudotime analysis. **C** Expression patterns of phenotypic markers (ACTA2, TAGLN, KLF4, RUNX2, S100A4, SPP1) along pseudotime, with violin plots comparing AD and healthy samples. **D** Heatmap showing gene expression dynamics across identified clusters and inferred cell fates (pre-branch, cell fate 1, cell fate 2). Pseudotime trajectory analysis was performed using Monocle. Differentially expressed genes along pseudotime or branch-dependent genes were identified using the statistical framework implemented in Monocle with multiple-testing correction where applicable

To characterize the differentiation dynamics of VSMC lineages and pinpoint the critical divergence between physiological and pathological states in AD, we reconstructed the developmental trajectory of VSMCs using pseudotime and state segmentation analysis. When stratified by AD and healthy groups, the pseudotime distribution patterns were largely similar between AD and Healthy samples before node 2, implying comparable temporal progression trends. However, after the branching at Node 1, cells from the AD group were significantly more abundant than those from the healthy group (Fig. 3A). This suggests that Node 1 serves as the critical bifurcation point where VSMC phenotypic switching occurs, leading to AD development. Additionally, the state segmentation (State 1 to 5) demonstrated that both AD and Healthy cells spanned multiple states along the pseudotime axis. Notably, a substantially higher proportion of cells in State 4 was observed in the AD group compared to the healthy group, suggesting that State 4 represents a disease-specific pathological state (Fig. 3A).

To investigate the expression dynamics of the four key genes, we analyzed the relative expression of *CASQ2*, *DES*, *ERRFI1*, and *FHL1* along the pseudotime axis, stratified by group (AD vs. Healthy) and cellular state (State 1–5) (Fig. 3B). For *CASQ2*, notable expression differences were observed across pseudotime, group, and state. Along pseudotime, *CASQ2* expression showed a distinct downward trend, with high expression concentrated in the early pseudotime stage. When stratified by Type (AD in red and Healthy in blue), AD samples exhibited higher *CASQ2* expression compared to Healthy samples, especially in the early pseudotime phase. Further segmentation by State (States 1–5) revealed that State 1 (red) had the highest *CASQ2* expression, while expression gradually decreased in States 2–5, indicating that *CASQ2* expression was most prominent in the early cellular state, particularly in the AD group.

To define the biological identity of these cellular states, we evaluated established VSMC phenotypic markers (Fig. 3C). Contractile markers, including ɑ-SMA (ACTA2) and SM22-ɑ (TAGLN), exhibited peak expression in the early pseudotime stages (State 1). However, their expression levels declined sharply as cells transitioned past Node 1 into State 4. Quantitative violin plots confirmed a significant overall downregulation of *TAGLN* and *ACTA2* in AD samples. Parallel to the decline of contractile markers, the synthetic marker *KLF4* and the osteochondrogenic marker SPP1 (Osteopontin) were significantly upregulated in the terminal trajectory stages (States 4 and 5). Notably, both markers were significantly overexpressed in the AD group relative to healthy tissues, reflecting a robust transition toward a synthetic and osteochondrogenic phenotype.

To elucidate the molecular mechanisms governing this divergence at Node 1, we categorized the top 100 branch-dependent differentially expressed genes into four functional clusters (Fig. 3D; Supplementary Fig. 2B). In Cluster 2, genes such as *CASQ2*,* ACTA2*,* TAGLN* and *MYH11* exhibited high expression. Functional enrichment for Cluster 2 was predominantly associated with “muscle contraction” and “vasoconstriction”, while KEGG analysis confirmed enrichment in “Vascular smooth muscle contraction” (Supplementary Fig. 2C-2D). Notably, *CASQ2* expression remained highly enriched alongside these canonical contractile markers. In addition to these canonical contractile programs, the CASQ2-containing cluster was also enriched in mitochondrial function-related terms, including “oxidative phosphorylation”, “mitochondrial respiratory chain complex assembly”, “electron transport chain”, “ATP metabolic process”, “mitochondrial inner membrane”, “mitochondrial matrix”, and “mitochondrial protein-containing complex”. These findings suggest a potential link among CASQ2-associated VSMC transition, calcium regulation, and mitochondrial metabolic remodeling. In contrast, Cluster 1 was characterized by the upregulation of pathological markers such as *SPP1*,* SPARC* and *VCAN*. Functional enrichment analysis revealed that this cluster is predominantly involved in ‘extracellular matrix organization’ and ‘ossification’, reflecting a transition toward a synthetic and osteochondrogenic phenotype. This reflects a shift toward a pathological synthetic and osteochondrogenic phenotype, accompanied by the progressive decline of *CASQ2*. In Cluster 4, enrichment was observed in “response to hypoxia” and “Fluid shear stress and atherosclerosis”, suggesting that the loss of *CASQ2* initiates a cascade triggering cellular stress, which likely exacerbates the fragility of the aortic wall.

Taken together, these findings demonstrate a strong reciprocal relationship between the loss of *CASQ2* and the activation of pathological VSMC remodeling. The progressive downregulation of *CASQ2* at Node 1 serves as a critical molecular signature of VSMC phenotypic switching, driving the transition from a stable contractile phenotype toward a detrimental synthetic, osteochondrogenic, and stress-induced phenotype that ultimately compromises aortic wall integrity in AD.

### CASQ2 restoration attenuated VSMC phenotypic switching and ER stress in vivo

**Fig. 4:**
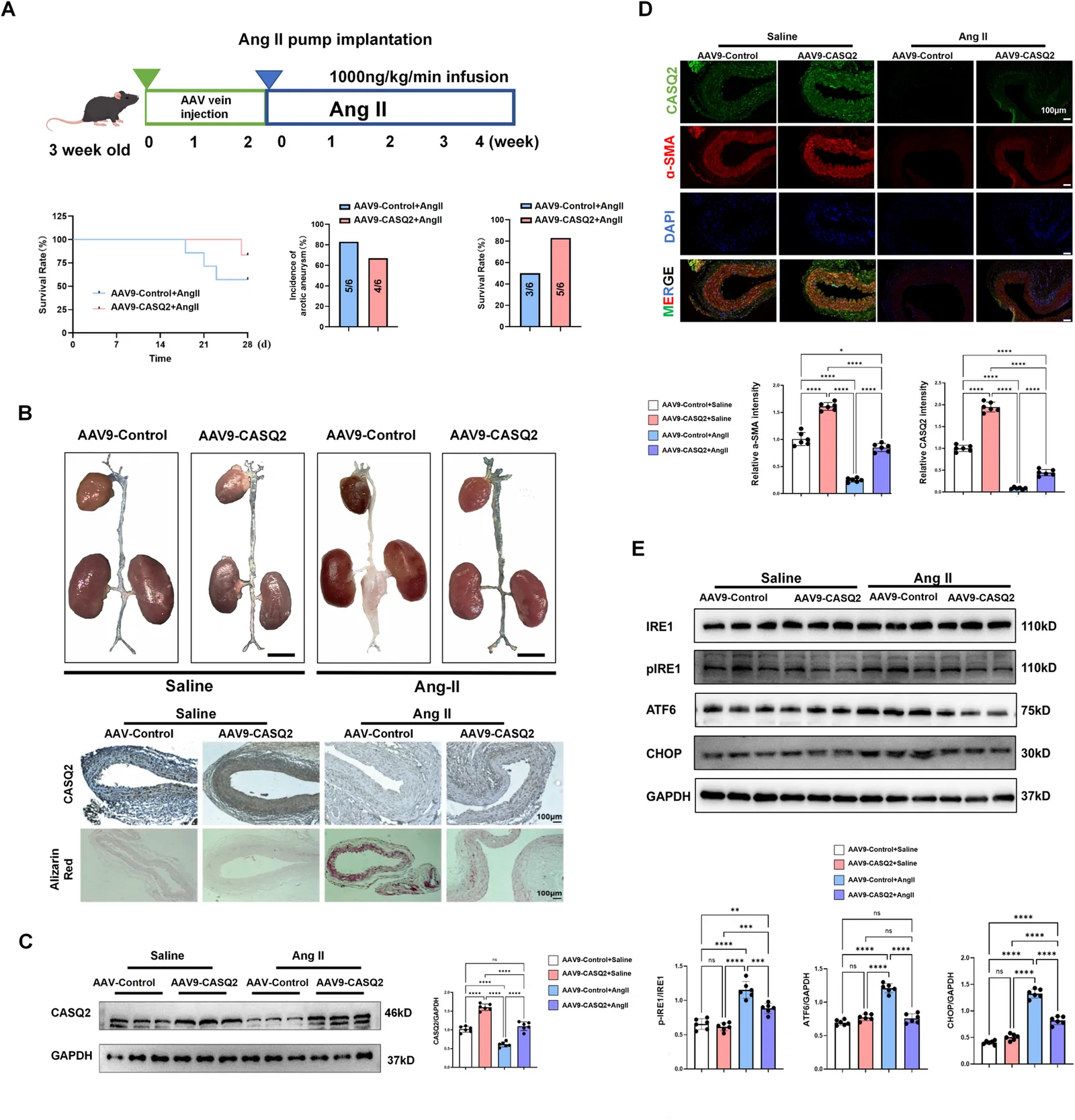
CASQ2 reduction aggravated the formation of VSMC phenotypic switching in vivo.**A** Schematic representation of the experimental timeline for AAV vein injection and subsequent treatments with Ang II. The Kaplan-Meier survival curve and bar plot of survival rates of AAV9-Control+AngII and AAV9-CASQ2 + AngII group. **B** Gross morphological images of aortas from mice treated with saline+AAV9-Control, Ang II+AAV9-CASQ2, saline + AAV9-CASQ2 or Ang II + AAV9-CASQ2. The scale bars = 1 cm. Immunohistochemistry for CASQ2 and Alizarin red staining of aortas under saline and Ang II treatment conditions. The scale bars = 100 μm. **C** Western blot analysis of CASQ2 expression in AAV9-Control and AAV9-CASQ2-treated mice under saline and Ang II conditions. **D** Immunofluorescence staining for CASQ2 (green), α-SMA (red), and DAPI (blue). Merged images show colocalization. Scale bar = 50 μm. **E** Western blot analysis of IRE1, pIRE1, ATF6, CHOP and GAPDH expression. Survival curves were analyzed using the Kaplan-Meier method and compared using the log-rank test. Comparisons among multiple groups were performed u-ing one-way ANOVA followed by Tukey’s post hoc test or the Kruskal-Wallis test followed by Dunn’s multiple-comparison test, as appropriate. Western blot bands were quantified by densitometry and normalized to GAPDH. The number of animals in each group is indicated in the figure or figure legend. (Data are presented as the mean ± SEM. *, *P* < 0.05; **, *P* < 0.01; ***, *P* < 0.001; ns, no significant difference.All P values were obtained via one-way ANOVA.) The number of mice in each group was as follows: AAV9-Control + saline, *n* = 6; AAV9-CASQ2 + saline, *n* = 6; AAV9-Control + Ang II, *n* = 6; and AAV9-CASQ2 + Ang II, *n* = 6. Survival curves were analyzed using the Kaplan-Meier method and compared using the log-rank test. For survival analysis, the number of mice was AAV9-Control + Ang II, *n* = 6, and AAV9-CASQ2 + Ang II, *n* = 6

To determine whether CASQ2 reduction contributes to AD progression in vivo, we established an Ang II-induced experimental AD model with AAV9-mediated CASQ2 overexpression (Fig. 4A). The validation focused on the abdominal/suprarenal aorta, where Ang II-induced lesions were consistently observed in our model, to determine whether CASQ2 restoration could attenuate VSMC phenotypic switching, vascular calcification, and ER stress activation. Successful CASQ2 overexpression was confirmed by Western blot analysis (Fig. 4C). Ang II infusion markedly reduced endogenous CASQ2 expression and was associated with increased mortality. Notably, CASQ2 overexpression improved survival compared with the AAV9-Control + Ang II group (83.3% [5/6] vs. 50.0% [3/6])., indicating a protective effect of CASQ2 in Ang II-induced experimental AD (Fig. 4A). CASQ2 overexpression showed a trend toward reduced aneurysm incidence from 83.3% [5/6] to 66.7% [4/6]. Morphological examination revealed pronounced suprarenal aortic dilation in Ang II-treated mice, whereas AAV9-mediated CASQ2 overexpression significantly attenuated aneurysmal enlargement (Fig. 4B). Consistently, Ang II infusion induced marked medial calcification, as demonstrated by enhanced Alizarin Red staining, which was substantially reduced following CASQ2 overexpression (Fig. 4B). These results suggest that CASQ2 plays a protective role against vascular calcification in AD.

At the molecular level, Ang II treatment increased expression of the osteochondrogenic marker RUNX2 and reduced the contractile marker ɑ-SMA, indicating VSMC phenotypic switching. These alterations were partially reversed by CASQ2 overexpression (Fig. 4C-D). Given the established link between calcium dysregulation and ER stress, we assessed ER stress activation and found that Ang II markedly increased the expression of IRE1, p-IRE1, ATF6, and CHOP, whereas CASQ2 overexpression significantly suppressed these responses (Fig. 4E). Collectively, these findings demonstrate that CASQ2 overexpression protects against Ang II–induced aortic degeneration by limiting VSMC osteochondrogenic transition, suppressing vascular calcification, and mitigating ER stress activation in vivo.

### CASQ2 reduction promoted Ang II–induced VSMC phenotypic switching through Ca^2+^-dependent ER stress in vitro

**Fig. 5:**
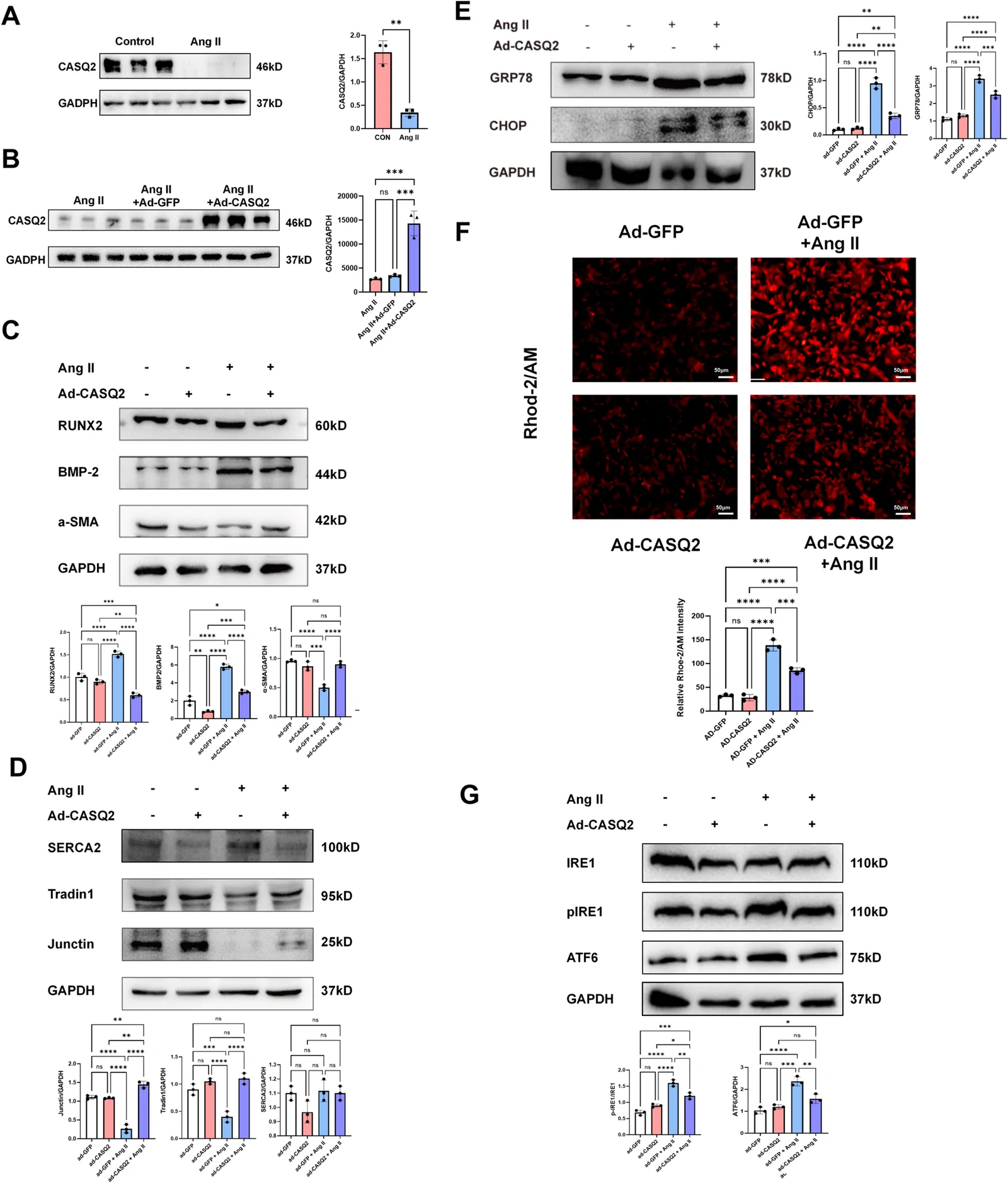
CASQ2 reduction promoted Ang II-induced VSMC phenotypic switching via Ca^2+^-dependent endoplasmic reticulum stress. **A**-**B** Western blot analysis of CASQ2 expression of VSMCs treated with Ang II (A) and Ang II-treated VSMCs infected with Ad-CASQ2 or Ad-GFP (B). **C** Western blot analysis of RUNX2, BMP-2, and α-SMA expression. **D** Western blot analysis of SERCA2, TRDN, and Junctin expression. **E** Western blot analysis of GRP78 and CHOP expression as markers of ER stress. **F** Representative images of the Ca^2+^ signal in VSMCs after Ang II treatment and quantification (*n* = 6 per group). Scale bar = 50 μm. **G **Western blot analysis of IRE1, pIRE1 and ATF6 expression. Comparisons among multiple groups were performed using one-way ANOVA followed by Tukey’s post hoc test or the Kruskal-Wallis test followed by Dunn’s multiple-comparison test, as appropriate. Western blot bands were quantified by densitometry and normalized to GAPDH. Rhod-2 fluorescence intensity was quantified after background subtraction and normalized to the control group to reflect relative intracellular Ca²⁺ levels. (Data are presented as the mean ± SEM. *, *P* < 0.05; **, *P* < 0.01; ***, *P* < 0.001; ****, *P* < 0.0001; ns, no significant difference. Statistical tests are described in the Methods.)

To further define the mechanistic role of CASQ2 in VSMC phenotypic switching, primary mouse VSMCs were stimulated with Ang II in vitro. Ang II treatment significantly reduced CASQ2 protein expression compared with control cells (Fig. 5A), recapitulating the in vivo findings. Consistent with phenotypic switching toward an osteochondrogenic state, Ang II markedly increased RUNX2 and BMP-2 expression while suppressing the contractile marker ɑ-SMA (Fig. 5B-C). Adenovirus-mediated CASQ2 overexpression (Ad-CASQ2) restored CASQ2 levels and significantly attenuated RUNX2 and BMP-2 induction, partially rescuing ɑ-SMA expression. These results indicate that CASQ2 restrains Ang II-induced osteochondrogenic transition of VSMCs.

Given the central role of CASQ2 in sarcoplasmic reticulum (SR) calcium buffering, we next examined key components of the SR calcium-release complex. Ang II significantly reduced Junctin and Triadin (TRDN) expression without affecting SERCA2 levels, suggesting that calcium dysregulation primarily involves altered SR release machinery rather than impaired calcium reuptake (Fig. 5D). CASQ2 overexpression restored Junctin and TRDN expression, supporting a stabilizing role of CASQ2 within the RyR2-associated calcium-release complex. To directly assess intracellular calcium homeostasis, Ca^2+^ imaging was performed using Rhod-2. Ang II stimulation markedly increased cytosolic Ca^2+^ levels, whereas Ad-CASQ2 significantly suppressed this elevation (Fig. 5F), indicating that CASQ2 reduction contributes to calcium overload in VSMCs. Because ER calcium depletion is a well-established trigger of unfolded protein response (UPR) activation, we evaluated ER stress markers. Ang II robustly induced GRP78 and CHOP expression and activated UPR signaling pathways, as evidenced by increased IRE1, p-IRE1, and ATF6 levels (Fig. 5E and G). CASQ2 overexpression markedly attenuated these responses, demonstrating that CASQ2 limits Ang II-induced ER stress activation.

Collectively, these data indicate that CASQ2 deficiency promotes Ang II-induced VSMC phenotypic switching by destabilizing SR calcium-release machinery, inducing intracellular Ca^2+^ overload, and triggering ER stress activation.

## Discussion

Acute AD remains a devastating cardiovascular emergency characterized by rapid medial destabilization and high early mortality despite advances in surgical intervention [1]. Although substantial progress has been made in identifying molecular mediators of aortic wall degradation—including matrix metalloproteinases, inflammatory cytokines, and extracellular matrix remodeling pathways—the upstream regulatory events that initiate VSMC dysfunction and medial failure remain incompletely defined [3, 12] Increasing evidence supports VSMC phenotypic switching as a central pathological feature of AD, linking contractile loss to structural instability [3, 10]. However, most prior transcriptomic studies rely on bulk sequencing of advanced lesions, potentially obscuring early and cell-specific regulatory transitions.

To address this limitation, we integrated WGCNA with single-cell RNA sequencing and pseudotime trajectory analysis. By intersecting transcriptomic profiles from AA and AD, two pathologies sharing a common substrate of medial degeneration, we delineated conserved regulatory modules associated with VSMC phenotypic switching. This integrative framework enabled us to isolate 64 candidate genes and ultimately identify *CASQ2* as a key regulatory node associated with VSMC transition in AD. Importantly, this strategy mitigates the confounding inflammatory noise characteristic of acute AD samples and strengthens the robustness of candidate selection. The WGCNA-based integration of AA and AD datasets should be interpreted as a strategy to identify conserved VSMC transition-related genes in aortic disease rather than AD-specific diagnostic biomarkers. Although AA and AD are distinct disease entities, both share medial degeneration, extracellular matrix remodeling and VSMC phenotypic switching. The additional AA-focused analysis showed that CASQ2 was also significantly reduced in AA samples compared with controls and displayed modest discriminatory performance, indicating that CASQ2 is not restricted to AD but may participate in broader aortic disease-related VSMC remodeling. Therefore, CASQ2 should be regarded as a VSMC transition-related functional candidate gene rather than an AD-specific biomarker. The novelty of this study lies in prioritizing CASQ2 from this conserved VSMC-related gene set and experimentally validating its role in AD-associated VSMC phenotypic switching, calcium dysregulation, and ER stress activation.

VSMC phenotypic plasticity represents a fundamental driver of aortic wall pathology. Loss of contractile markers such as *ACTA2* and *TAGLN*, coupled with induction of synthetic and osteochondrogenic regulators including *KLF4*,* RUNX2*, and *BMP2*, has been implicated in both aneurysm and dissection progression [3, 10]. In our study, *CASQ2* expression declined as AD progressed, paralleling loss of contractile identity. Angiotensin II (Ang II) stimulation reproduced this downregulation and induced osteochondrogenic marker expression, consistent with previous reports that Ang II promotes VSMC phenotypic modulation and medial degeneration [18]. Restoration of CASQ2 expression attenuated this phenotypic switching, suggesting that CASQ2 contributes to maintaining VSMC contractile phenotype. Although enrichment of contractile, cytoskeletal, and calcium-related pathways is consistent with known VSMC biology, these findings validate the biological relevance of our WGCNA and trajectory-based screening strategy rather than constituting the main novelty of the study. The key novel aspect is the identification of CASQ2 as a VSMC-enriched functional candidate gene within conserved aortic disease-associated transition modules and the experimental demonstration that CASQ2 restoration attenuates calcium dysregulation, ER stress activation, and osteochondrogenic VSMC switching in AD-related models.

Mechanistically, *CASQ2* is a luminal Ca^2+^-binding protein that regulates ryanodine receptor 2 (RyR2)-mediated calcium release within the SR [19, 20]. Beyond buffering, *CASQ2* functions as a structural scaffold coordinating the RyR2–junctin–triadin complex. Disruption of this complex promotes diastolic Ca^2+^ leak and cytosolic calcium elevation. Our data demonstrate that AD and Ang II exposure reduce CASQ2, junctin, and triadin expression, whereas SERCA2 levels remain unchanged, indicating that calcium dysregulation primarily involves altered SR release rather than impaired reuptake. AAV9-mediated CASQ2 restoration normalized junctin and triadin expression, supporting a stabilizing role of CASQ2 in SR calcium-release machinery.

Calcium dysregulation has well-established links to VSMC phenotypic modulation and vascular calcification [21, 22]. Elevated intracellular Ca^2+^ can activate osteochondrogenic transcription programs and disrupt cellular proteostasis. Importantly, perturbations in ER/SR calcium homeostasis trigger the unfolded protein response (UPR), as ER-resident chaperones require high luminal calcium for proper protein folding [22]. In our model, CASQ2 reduction was accompanied by robust activation of ER stress markers (GRP78, CHOP, IRE1, ATF6). ER stress has been shown to drive VSMC phenotypic switching and vascular remodeling [19]. Restoration of CASQ2 mitigated ER stress activation and suppressed osteochondrogenic differentiation, suggesting that calcium-dependent ER stress represents a downstream pathological cascade linking CASQ2 loss to VSMC transition. The potential clinical implications of calcium-handling pathways in aortic disease should be interpreted cautiously. Calcium channel blockers are commonly used antihypertensive agents; however, previous experimental work in Marfan syndrome reported that calcium channel blockade accelerated aortic aneurysm expansion, rupture, and premature lethality through mechanisms involving ERK1/2 and angiotensin II type 1 receptor signaling [23]. Although the present study did not directly examine calcium channel blockers or antihypertensive treatment, these findings highlight the complexity of calcium signaling in aortic wall homeostasis. Our results support the concept that disturbed intracellular calcium handling, rather than calcium modulation, may contribute to VSMC phenotypic switching and aortic remodeling. Therefore, the current findings should not be interpreted as evidence for or against calcium channel blocker use in patients with aortic disease, but rather as mechanistic support for the importance of calcium homeostasis in maintaining VSMC stability.

CASQ2 has rarely been investigated in the context of aortic disease. A previous genetic study reported CASQ2-related enrichment in aorta artery expression and placed CASQ2 within a cardiovascular gene interaction network, suggesting a potential link between CASQ2 and cardiovascular calcium-handling biology [24]. In addition, recent bioinformatic work in Stanford type A aortic dissection emphasized the involvement of endoplasmic reticulum stress-related genes and immune infiltration in disease pathogenesis [25]. These studies provide genetic, bioinformatic, or pathway-level support for the relevance of CASQ2-related calcium handling and ER stress biology in cardiovascular and aortic disease. The present study extends these observations by identifying CASQ2 as a VSMC-enriched functional candidate gene and experimentally demonstrating that CASQ2 restoration attenuates calcium dysregulation, ER stress activation, and osteochondrogenic VSMC transition.

Mitochondrial dysfunction may represent another potential mechanism linking CASQ2-mediated calcium dysregulation to VSMC phenotypic switching. Recent studies have increasingly highlighted the contribution of mitochondrial metabolic remodeling, impaired oxidative phosphorylation, mitochondrial ROS generation, and altered mitochondrial dynamics to aneurysm/dissection formation and VSMC phenotypic modulation [26, 27]. In our bioinformatic analysis, the CASQ2-containing pseudotime trajectory cluster was enriched not only in VSMC contractile and calcium-related processes, but also in mitochondrial function-related terms, including oxidative phosphorylation, mitochondrial respiratory chain complex assembly, electron transport chain, ATP metabolic process, mitochondrial inner membrane, mitochondrial matrix, and mitochondrial protein-containing complex. Given the close coupling between SR/ER calcium release and mitochondrial calcium uptake, CASQ2 loss may indirectly affect mitochondrial calcium homeostasis, oxidative phosphorylation, and ROS generation, thereby amplifying pathological VSMC remodeling [28, 29]. However, mitochondrial respiration, mitochondrial Ca²⁺ dynamics, mitochondrial membrane potential, and mitochondrial ROS production were not directly assessed in the present study. Future studies are needed to determine whether CASQ2 regulates VSMC phenotypic switching through coordinated SR/ER–mitochondrial calcium signaling.

## Limitations

Because the initial candidate genes were identified from overlapping AA and AD modules, the present study does not establish AD specificity. CASQ2 should therefore be regarded as a candidate VSMC functional regulator involved in aortic disease-related remodeling rather than an AD-specific diagnostic biomarker. Although the additional AA-focused analysis provides useful context, larger matched AA and AD cohorts are needed to determine whether CASQ2 has disease-specific or broadly shared roles across aortic diseases. Although the Ang II-induced experimental AD model recapitulates several pathological features relevant to AD, including aortic dilation, medial remodeling, VSMC phenotypic switching, and vascular calcification, it does not fully reproduce the anatomical, hemodynamic, and age-related complexity of human AD. In particular, the use of 3-week-old mice may limit direct extrapolation to adult or elderly patients with AD. Therefore, the protective effects of CASQ2 observed in this model should be interpreted cautiously and further validated in additional AD models and human AD tissues. An anatomical mismatch existed among the discovery and validation datasets. The human AD datasets mainly involved ascending/thoracic aortic tissues, whereas the AA datasets and experimental validation were largely based on abdominal or suprarenal aortic tissues. Thus, the present study supports a conserved VSMC transition-related mechanism but does not establish segment-specific effects of CASQ2 across the entire aorta. Future studies should validate CASQ2 function in matched thoracic and abdominal aortic tissues and segment-specific animal models. Finally, although the CASQ2-containing pseudotime trajectory cluster was enriched in mitochondrial function-related terms, mitochondrial respiration, mitochondrial Ca²⁺ dynamics, mitochondrial membrane potential, and mitochondrial ROS production were not directly assessed. Therefore, the potential involvement of mitochondrial dysfunction in CASQ2-mediated VSMC phenotypic switching requires further investigation.

## Conclusion

Taken together, our findings support a model in which CASQ2 loss destabilizes SR calcium-release machinery, leading to calcium imbalance, ER stress activation, and subsequent osteochondrogenic phenotypic switching. While additional upstream regulatory factors likely contribute to AD pathogenesis, our integrative multi-omic approach identifies CASQ2 as a critical regulatory component of this axis. By uncovering a CASQ2–SR/ER stress signaling pathway in VSMCs, this study provides mechanistic insight into medial failure and highlights intracellular calcium homeostasis as a potential therapeutic target for limiting AD progression.

## Supplementary Information

Supplementary Material 1.

Supplementary Material 2.

## Data Availability

The data supporting the conclusions of this research are available from the corresponding author upon reasonable request.
