# Supplementary material for: Loss of CASQ2 promotes vascular smooth muscle cell phenotypic switching in aortic dissection uncovered by integrated single-cell transcriptomics
Source: BMC Med Genomics. 2026 Jun 13;19:134. doi: 10.1186/s12920-026-02410-w (PMC13491616; doi:10.1186/s12920-026-02410-w)

Full unedited gel for Figure 4C

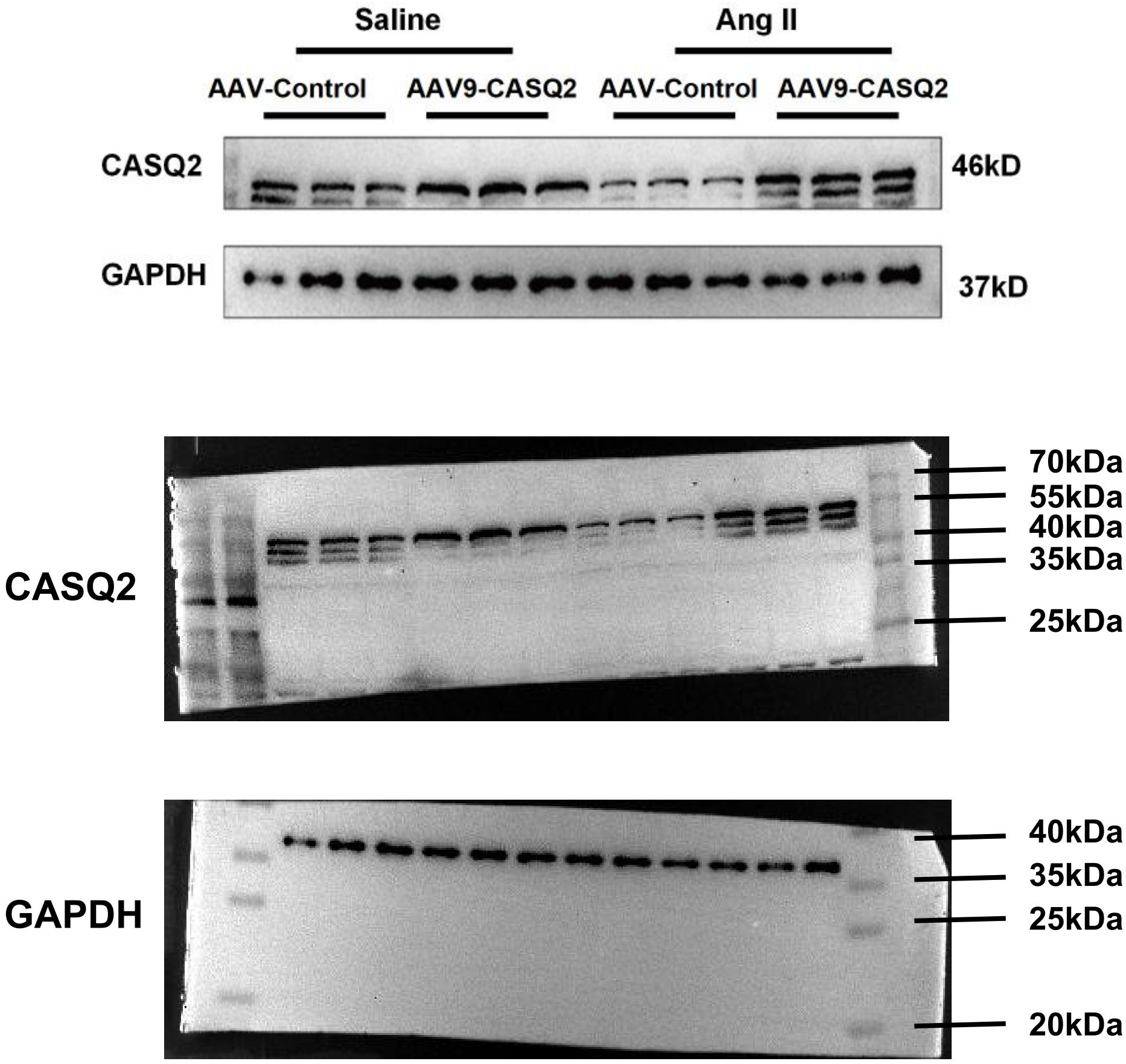

Full unedited gel for Figure 4E

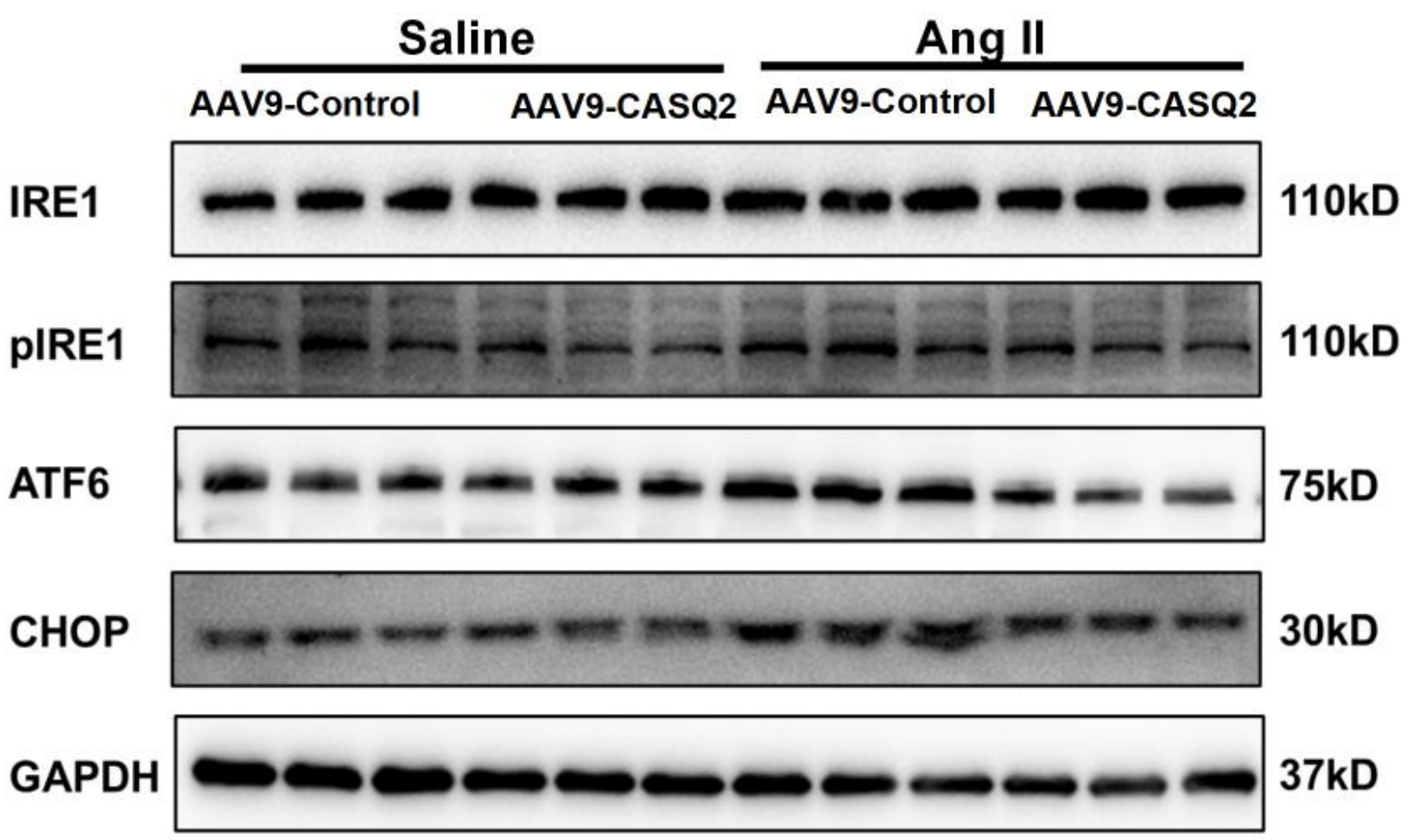

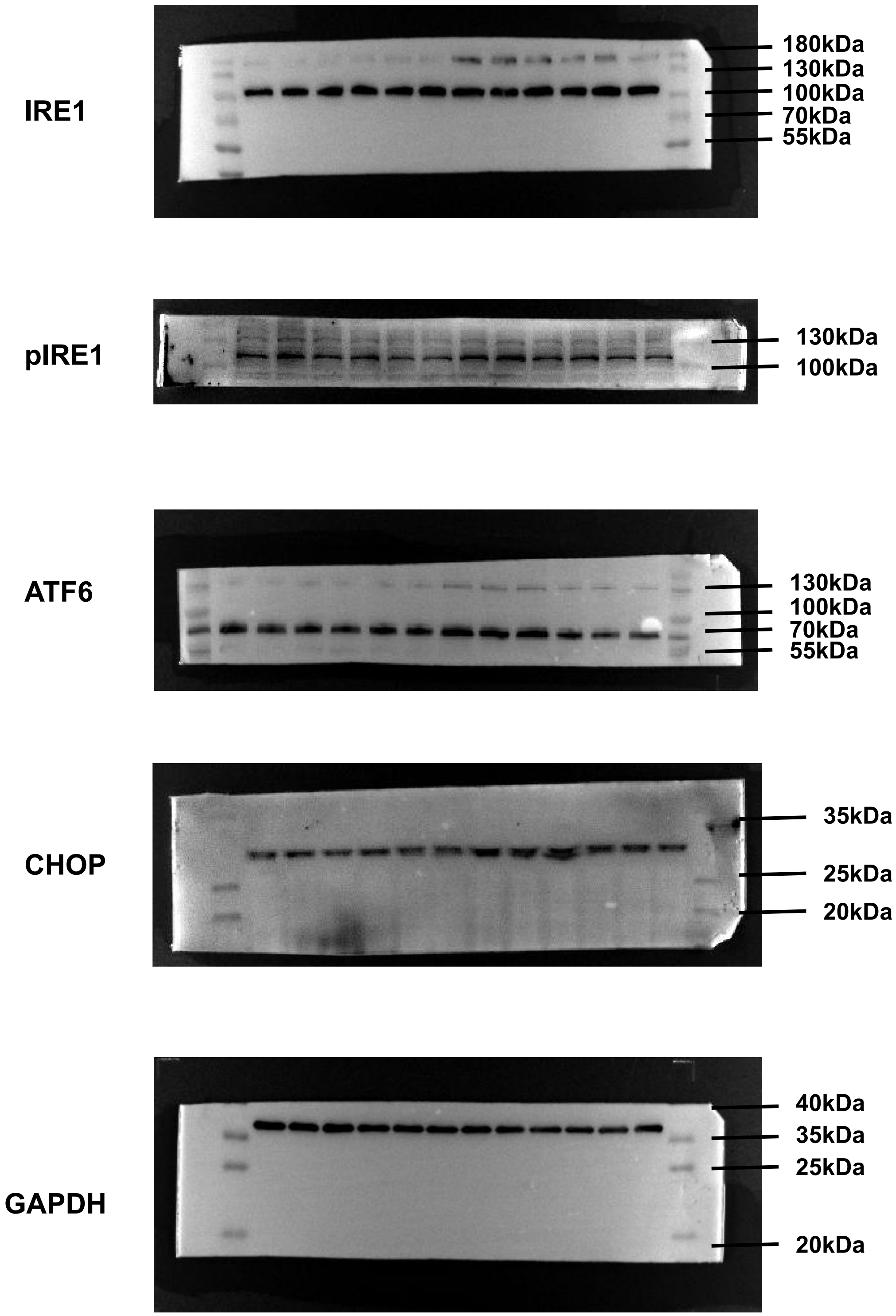
IRE1
180kDa
130kDa
100kDa
70kDa
55kDa
pIRE1
130kDa
100kDa
ATF6
130kDa
100kDa
70kDa
55kDa
CHOP
35kDa
25kDa
20kDa
GAPDH
40kDa
35kDa
25kDa
20kDa

Full unedited gel for Figure 5A

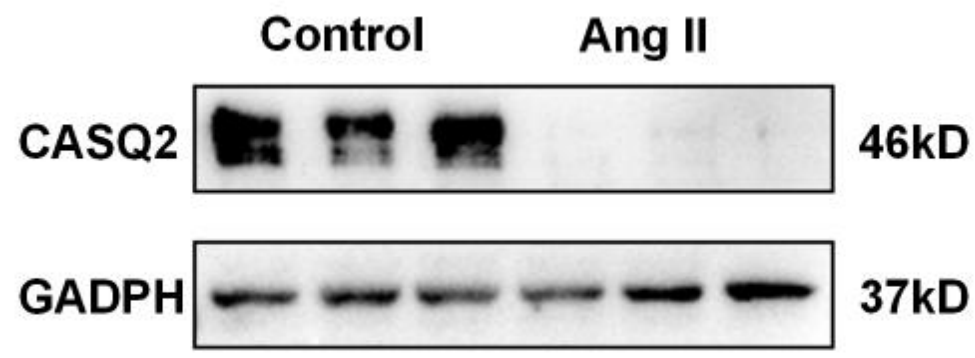

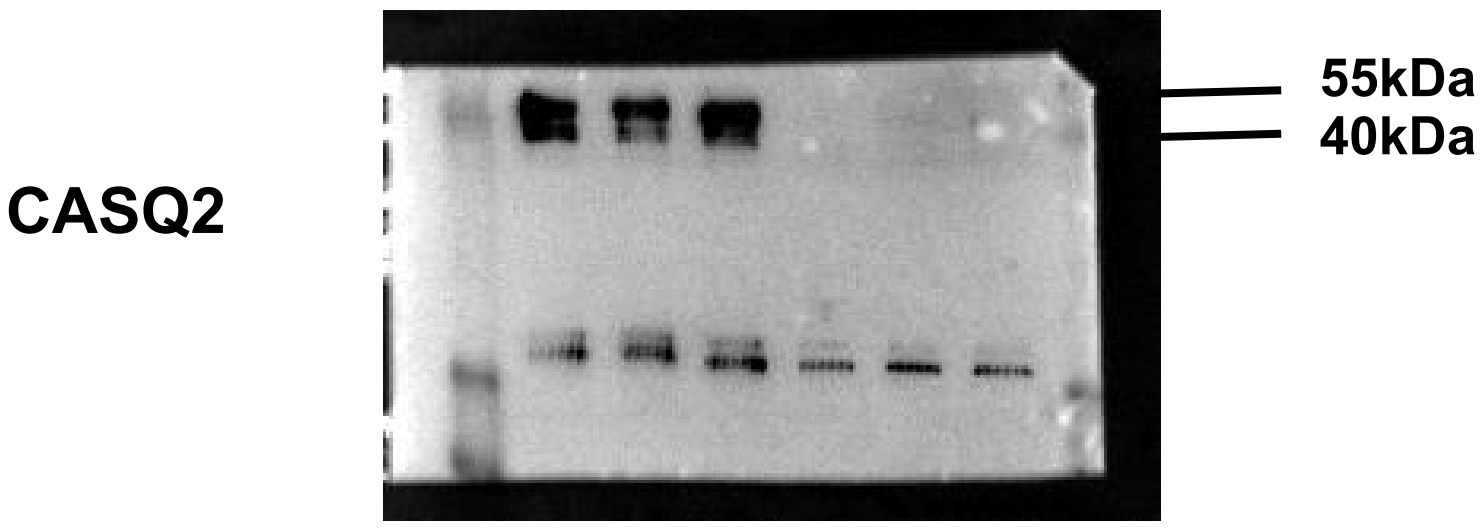

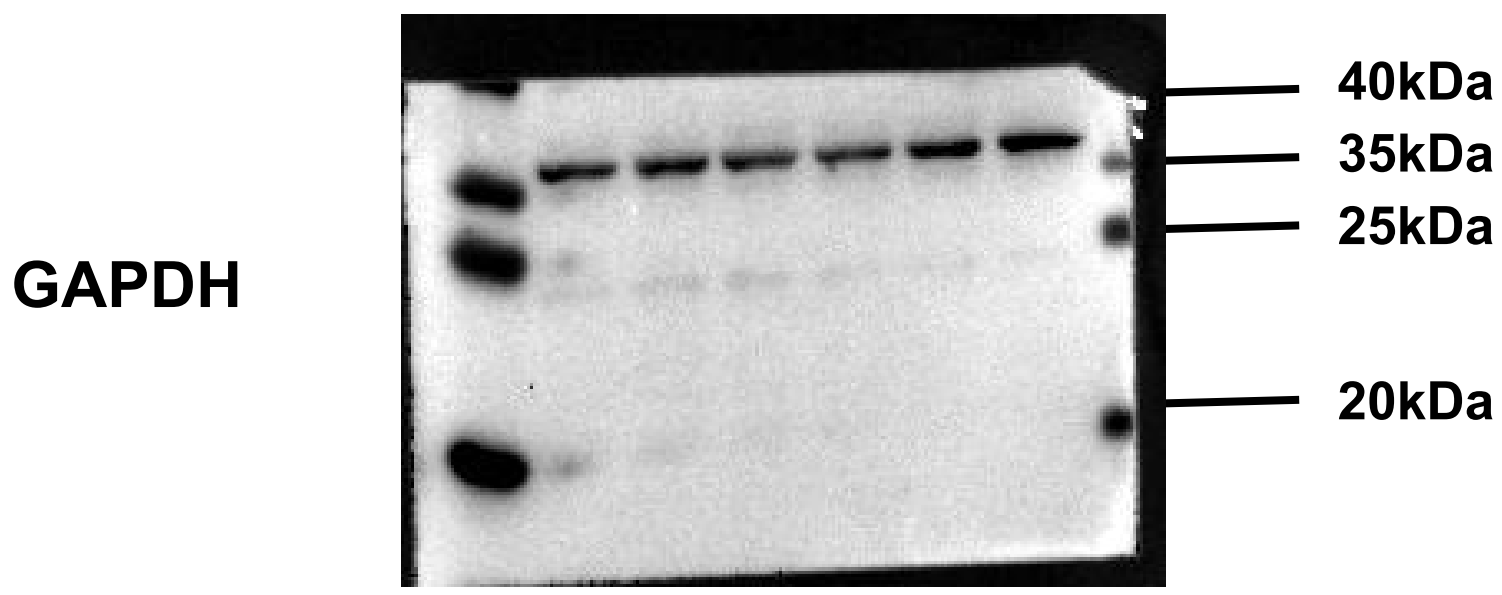

Full unedited gel for Figure 5B

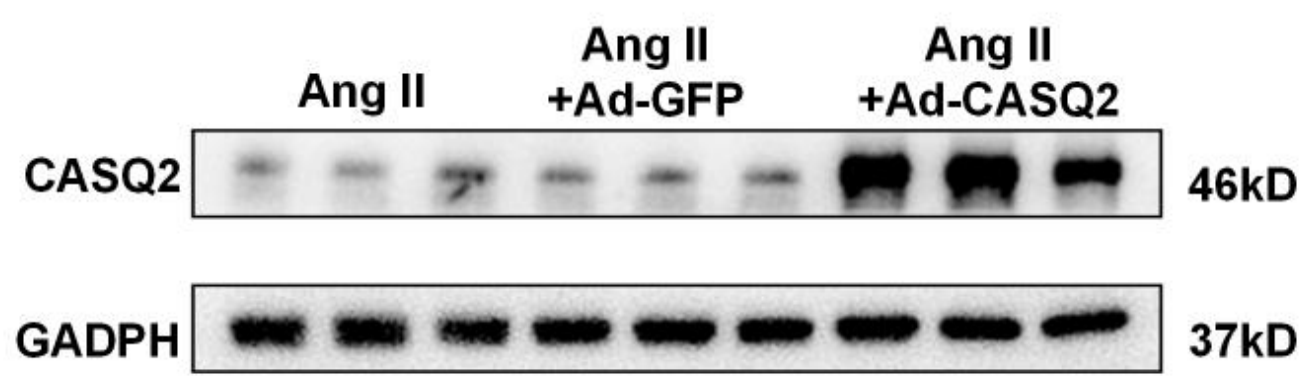

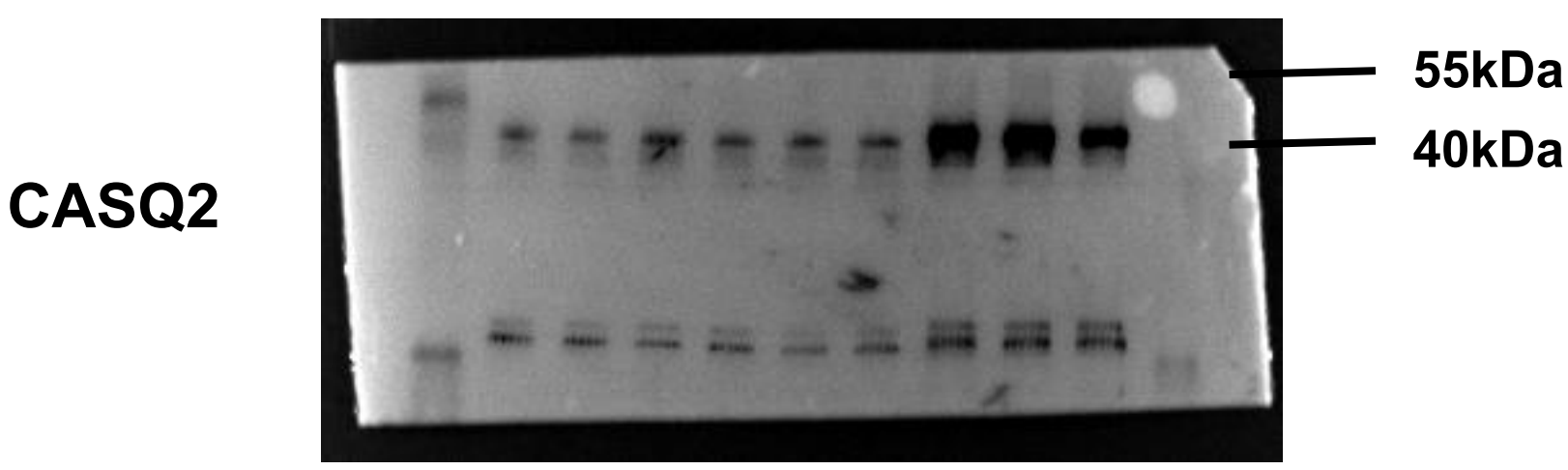

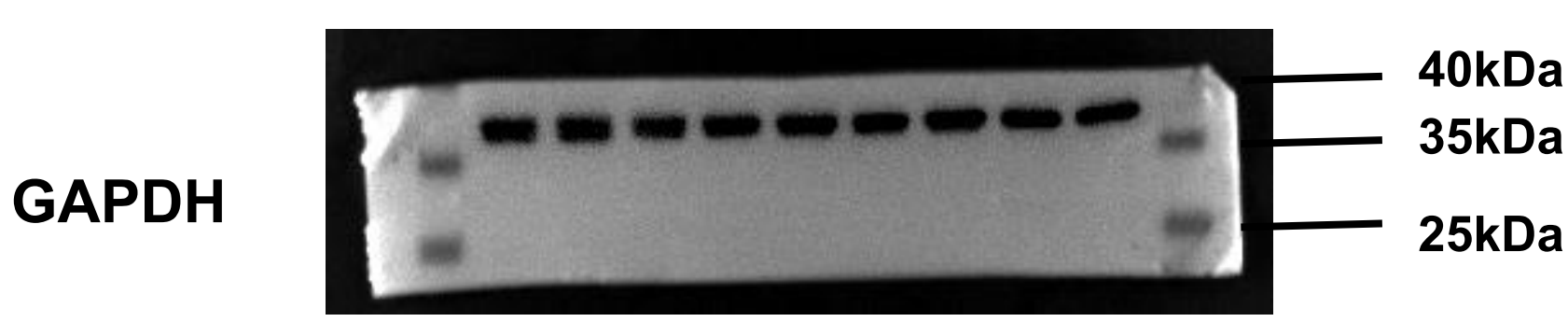

Full unedited gel for Figure 5C

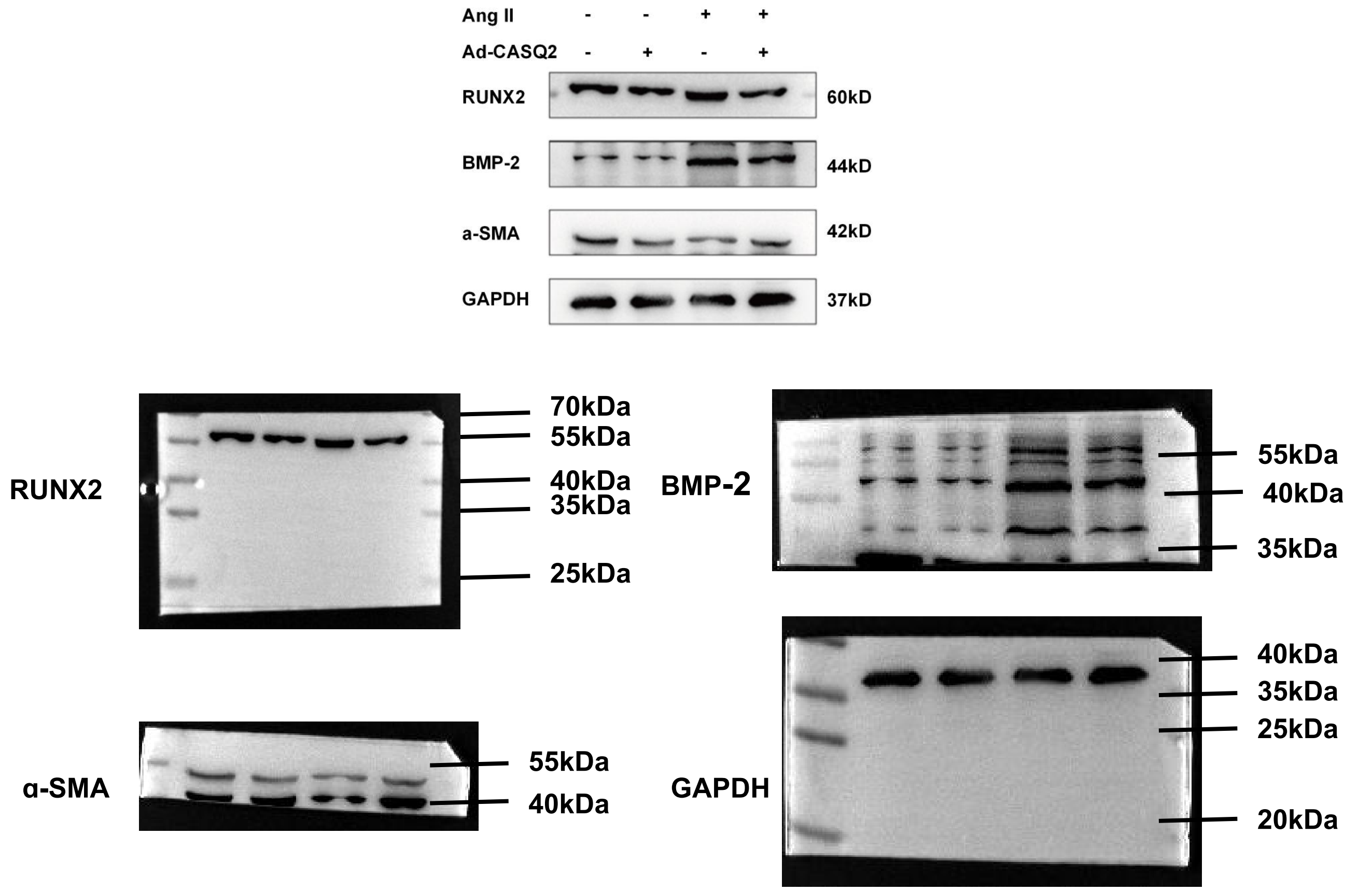

Full unedited gel for Figure 5D

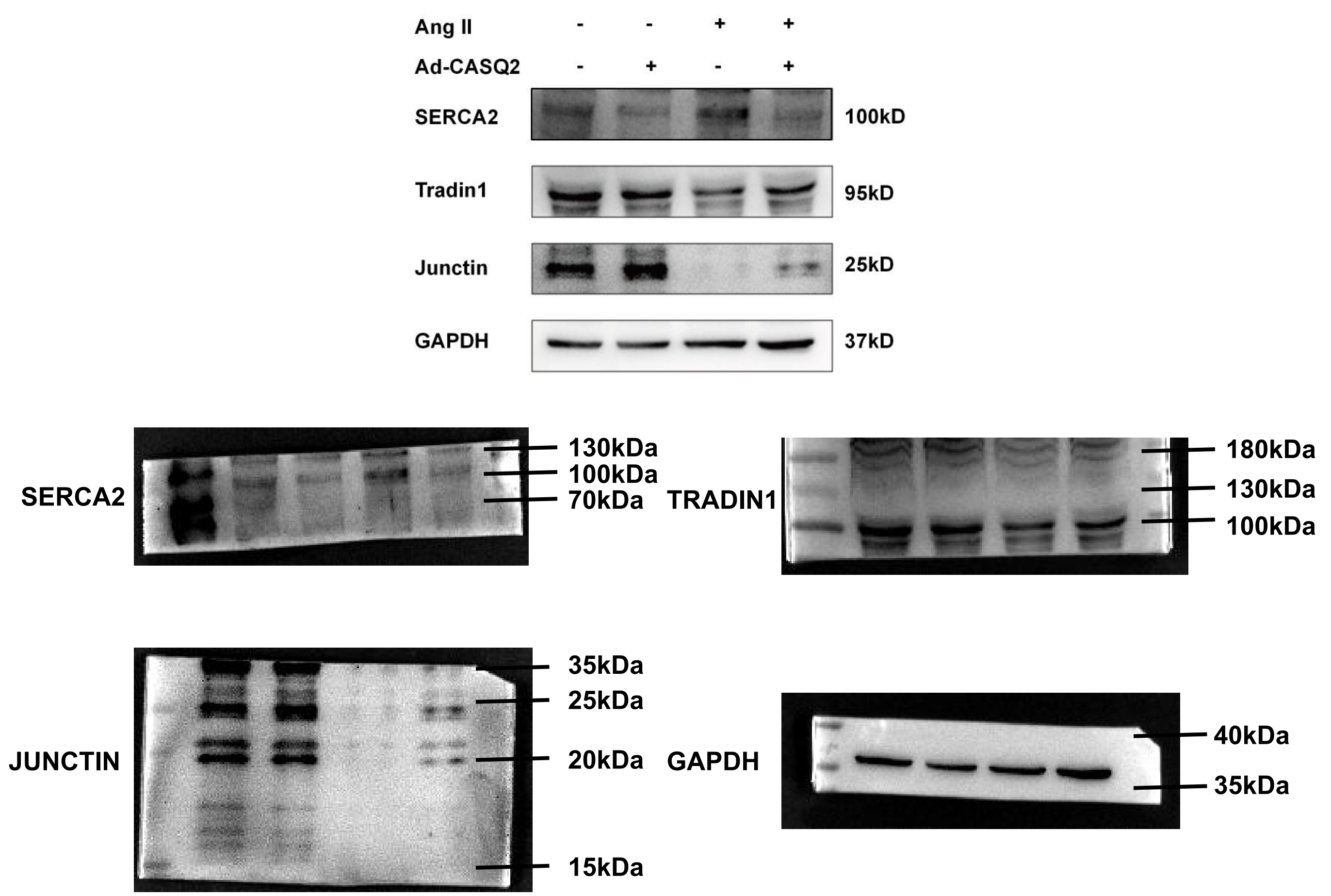

Full unedited gel for Figure 5E

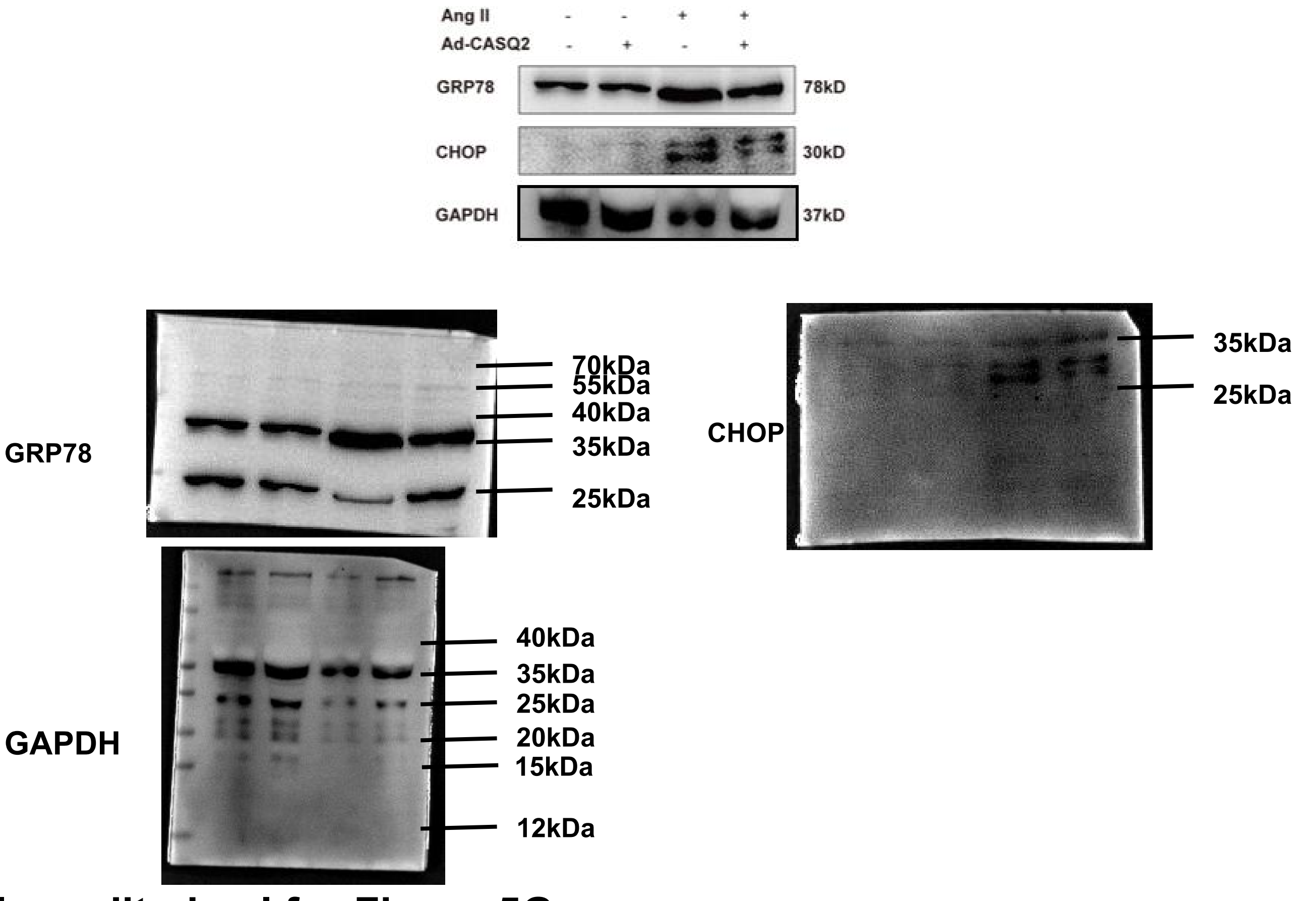

Full unedited gel for Figure 5G

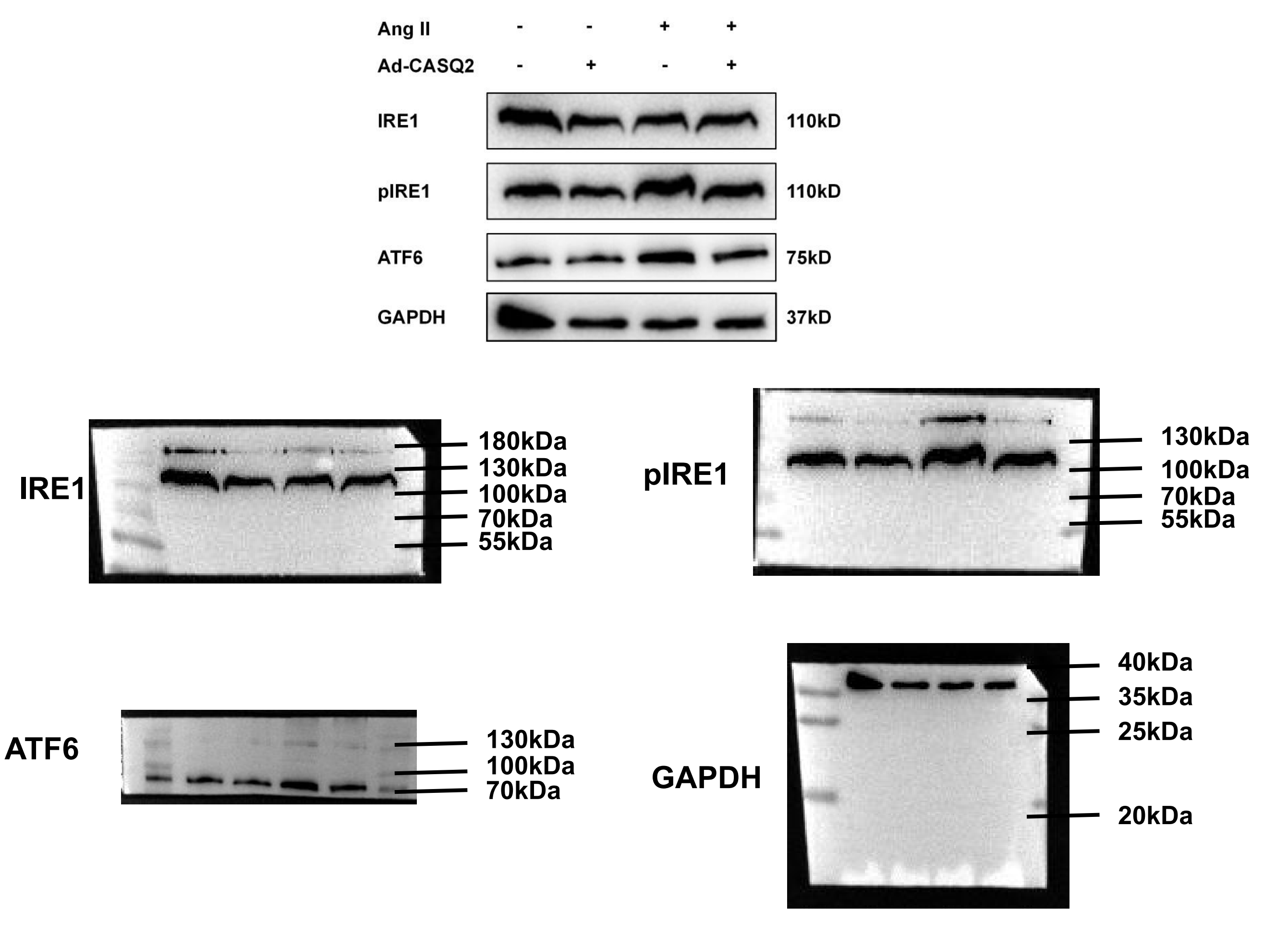

Supplement: Supplementary file 1 [file 12920_2026_2410_MOESM2_ESM.pdf]
