# Supplementary material for: Loss of CASQ2 promotes vascular smooth muscle cell phenotypic switching in aortic dissection uncovered by integrated single-cell transcriptomics
Source: BMC Med Genomics. 2026 Jun 13;19:134. doi: 10.1186/s12920-026-02410-w (PMC13491616; doi:10.1186/s12920-026-02410-w)
Supplement: Supplementary file 2 [file 12920_2026_2410_MOESM1_ESM.docx]

**Online Supplementary Material**

**Loss of CASQ2 promotes vascular smooth muscle cell phenotypic switching in aortic dissection uncovered by integrated single-cell transcriptomics**

**Supplementary Figure 1.** (A) KEGG enrichment analysis of AA module blue genes and AD module turquoise genes. (B) GO and KEGG enrichment analysis of AA moudule turquoise genes. (C) GO and KEGG enrichment analysis of AD module genes.

**Supplementary Figure 2.** (A) The subplot shows the average expression and presence of gene expression across various cell types, including B lymphocyte, Monocyte, T lymphocyte, Macrophage, Smooth Muscle Cell, Astrocyte, and Endothelial. The subplot compares the cell type composition between AD and Healthy samples, with different colors representing distinct cell types. (B) A scatter plot depicting the relationship between two gene expression metrics, with a grey trend line highlighting the general correlation pattern. (C-D) GO and KEGG enrichment analysis on genes of the 4 clusters in heatmap shown in Figure 3C.

**Supplementary Figure 3. (A)** Boxplots showing the expression levels of four candidate genes, CASQ2, DES, ERRFI1, and FHL1, in control and aortic aneurysm (AA) samples. CASQ2, ERRFI1, and FHL1 were significantly downregulated in AA samples compared with controls, whereas DES showed a nonsignificant downward trend. (B) Receiver operating characteristic (ROC) curve analysis evaluating the discriminatory performance of the four candidate genes in AA. The AUC values were 0.652 for CASQ2, 0.620 for DES, 0.683 for ERRFI1, and 0.713 for FHL1. Data are presented as boxplots with individual data points. *P < 0.05; **P < 0.01; ns, not significant. AA, aortic aneurysm; AUC, area under the curve; Con, control; ROC, receiver operating characteristic.
